# A Genome-Wide Analysis Reveals Stress and Hormone Responsive Patterns of TIFY Family Genes in *Brassica rapa*

**DOI:** 10.3389/fpls.2016.00936

**Published:** 2016-06-28

**Authors:** Gopal Saha, Jong-In Park, Md. Abdul Kayum, Ill-Sup Nou

**Affiliations:** Department of Horticulture, Sunchon National UniversitySuncheon, South Korea

**Keywords:** *TIFY*, *JAZ*, abiotic and biotic stress, hormone, gene expression, *Brassica rapa*, Chiifu, Kenshin

## Abstract

The TIFY family is a plant-specific group of proteins with a diversity of functions and includes four subfamilies, *viz*. ZML, TIFY, PPD, and JASMONATE ZIM-domain (JAZ) proteins. TIFY family members, particularly JAZ subfamily proteins, play roles in biological processes such as development and stress and hormone responses in *Arabidopsis*, rice, chickpea, and grape. However, there is no information about this family in any *Brassica* crop. This study identifies 36 *TIFY* genes in *Brassica rapa*, an economically important crop species in the *Brassicaceae*. An extensive *in silico* analysis of phylogenetic grouping, protein motif organization and intron-exon distribution confirmed that there are four subfamilies of BrTIFY proteins. Out of 36 *BrTIFY* genes, we identified 21 in the JAZ subfamily, seven in the TIFY subfamily, six in ZML and two in PPD. Extensive expression profiling of 21 *BrTIFY JAZs* in various tissues, especially in floral organs and at different flower growth stages revealed constitutive expression patterns, which suggest that *BrTIFY JAZ* genes are important during growth and development of *B. rapa* flowers. A protein interaction network analysis also pointed to association of these proteins with fertility and defense processes of *B. rapa*. Using a low temperature-treated whole-genome microarray data set, most of the *JAZ* genes were found to have variable transcript abundance between the contrasting inbred lines Chiifu and Kenshin of *B. rapa*. Subsequently, the expression of all 21 *BrTIFY JAZs* in response to cold stress was characterized in the same two lines via qPCR, demonstrating that nine genes were up-regulated. Importantly, the *BrTIFY JAZs* showed strong and differential expression upon JA treatment, pointing to their probable involvement in JA-mediated growth regulatory functions, especially during flower development and stress responses. Additionally, *BrTIFY JAZs* were induced in response to salt, drought, *Fusarium*, ABA, and SA treatments, and six genes (*BrTIFY3a, 3b, 6a, 9a, 9b*, and *9c*) were identified to have co-responsive expression patterns. The extensive annotation and transcriptome profiling reported in this study will be useful for understanding the involvement of *TIFY* genes in stress resistance and different developmental functions, which ultimately provides the basis for functional characterization and exploitation of the candidate *TIFY* genes for genetic engineering of *B. rapa*.

## Introduction

The TIFY gene family is plant-specific and encodes putative transcription factors (TFs) characterized by a highly conserved motif (TIF[F/Y]XG) positioned within an approximately 28-amino-acid TIFY domain (Vanholme et al., 2007; Bai et al., 2011). *ZIM* (*ZINC-FINGER EXPRESSED IN INFLORESCENCE MERISTEM*) from *Arabidopsis* was the first identified member of TIFY gene family (Nishii et al., 2000). Based on phylogenetic and structural analyses, genes of this family can be divided into four groups. The TIFY subfamily proteins contain only the TIFY domain (Staswick, 2008), whereas ZIM and ZIM-like (ZML) proteins, together forming the ZML subfamily, also contain both a C2C2-GATA zinc-finger DNA-binding domain and a CCT domain (CONSTANS, CO-like, TOC1). The Jasmonate ZIM-domain (JAZ) subfamily proteins contain a conserved Jas motif of approximately 27 amino acids near the C-terminus in addition to TIFY domains. These Jas sequences have similarity to the N-terminal portion of the CCT domain, with the characteristic motif SLX2FX2KRX2RX5PY (Staswick, 2008; Chung et al., 2009). By contrast, PEAPOD (PPD) subfamily proteins possess a characteristic N-terminal PPD domain and a modified Jas motif that lacks the conserved PY (Proline-Tyrosine) at their C-terminal region (Chung et al., 2009).

To date, most of the functionally validated *TIFY* genes are from *Arabidopsis*. So far, 18 *TIFY* genes in *Arabidopsis*, 22 in rice, 22 in poplar, 19 in grape, 30 in apple, and 34 in *Glycine soja* (wild soybean) have been identified (Vanholme et al., 2007; Ye et al., 2009; Zhang et al., 2012; Zhu et al., 2013; Li et al., 2015). *TIFY* genes are involved in the regulation of diverse aspects of plant development and responses to abiotic stresses and phytohormone treatments. For instance, overexpression of *ZIM/AtTIFY1* in *Arabidopsis* produced elongated petioles and hypocotyls (Shikata et al., 2004), while *AtTIFY4a* (*PPD1*) and *AtTIFY4b* (*PPD2*) have been reported to be involved in the synchronization of leaf growth (White, 2006). Among the TIFY family proteins, JAZ subfamily members are the best characterized and play pivotal roles in the jasmonic acid (JA) pathway (Chini et al., 2007; Thines et al., 2007; Yan et al., 2007). JA and its bioactive derivatives have been identified as prime factors in plant responses to both biotic (e.g., wounding, pathogen infection, and insect attack) and abiotic (e.g., drought, low temperature, salinity, ozone exposure) stresses (Devoto and Turner, 2005). *AtTIFY10b*/*JAZ1* is an important player in JA signal transduction. To stop their repression of JA-response genes, *JAZ1* is degraded in the presence of JA through the SCF^COI1^-dependent 26S proteasome pathway (Thines et al., 2007). JAZ proteins in different studies have been identified as repressors of JA-regulated transcription in *Arabidopsis*. Specifically, in the absence of JA, JAZ proteins interact with and repress the basic helix-loop-helix (bHLH) TFs MYC2 and MYC3. These TFs bind directly to DNA sequences and regulate the expression of downstream JA-responsive genes (Chini et al., 2007; Thines et al., 2007). *JAZ* genes show differential induction in response to JA treatment, herbivory, wounding, *Pseudomonas syringae* infection, and environmental stresses such as drought, low temperature and salinity (Chung et al., 2008; Ye et al., 2009; Demianski et al., 2012). *JAZ* genes have also been induced in response to different nutritional stress like phosphorus and bicarbonate (Aparicio-Fabre et al., 2013; Zhu et al., 2013). A more recent report on the involvement of *JAZ* genes against nutritional deficiency revealed their specificity with different nutrient stimuli (macro and micro nutrients) in rice and chickpea, where majority of the *JAZ* genes were induced by potassium (K) deficiency (Singh et al., 2015).

*Brassica* is a diversified crop genus that provides oil, vegetables, condiments, dietary fiber, and vitamin C (Talalay and Fahey, 2001). *Brassica rapa*, one of the most important members of the *Brassica* genus, has several subspecies and is a widely used vegetable in Asia. Over the course of evolution, the *Brassicaceae* genome experienced two whole-genome duplication (WGD) events as *Arabidopsis* beta and *Arabidopsis* alpha (Bowers et al., 2003). WGD results in gene duplication that is typically followed by significant gene loss (Lee et al., 2013). Evolutionary data indicate that *B. rapa* ssp. *pekinensis* is closely related to *Arabidopsis* and that since its divergence from *Arabidopsis* 13 to 17 Mya (Million years ago) it has experienced a whole-genome triplication (Town et al., 2006; Wang et al., 2011). This lineage thus provides a model for studying gene fractionation (Tang and Lyons, 2012; Kim et al., 2013). *B. rapa* as a representative of the *Brassica* “A” genome has been used as the model species and therefore, was selected for genome sequencing [*Brassica* Genome Gateway (http://brassica.nbi.ac.uk/); The Korea *Brassica rapa* Genome Project (http://www.brassica-rapa.org/BRGP/index.jsp)]. This species has a relatively small genome [approximately 529 megabase pairs (Mbp)] compared to other *Brassica* species and has proved a useful model for studying polyploidy.

Though, TIFY family genes have been investigated in *Arabidopsis*, which is a close relative of *B. rapa*, we set out to study this gene family in the relatively complex and diverse genome of *B. rapa*. The recent completion of the *B. rapa* Chiifu-401-42 genome sequencing (Wang et al., 2011) enabled us to carry out genome-wide research on TIFY proteins in *B. rapa*. We identified 36 *TIFY* genes in *B. rapa*. The putative *BrTIFY* genes were classified into four subfamilies and their gene structures, chromosomal locations, and potential gene duplicates were subsequently analyzed. From a low temperature-treated microarray data set, responses of all *BrTIFY* genes were investigated, comparing their expression between two contrasting inbred lines Chiifu and Kenshin of *B. rapa*. We further analyzed the responses of the *BrTIFY JAZ* genes to cold, salt, drought, *Fusarium oxysporum*, ABA, JA, and SA via quantitative RT-PCR. In an organ expression study, all the *BrTIFY JAZ* genes showed constitutive expression during the flower development stages and these results highlight the need for further study into the role(s) of *BrTIFY JAZ* genes in the development of *B. rapa* flowers.

## Materials and methods

### Identification of *B. rapa TIFY* genes

A SWISSPROT search on the *Brassica* database (BRAD) was conducted using keyword “TIFY” and a blast search using characteristic TIFY domain sequences was also employed in the BRAD to predict *B. rapa* TIFY proteins (V 1.5.tar.gz; http://brassicadb.org/brad/; Cheng et al., 2011). CDS and Protein sequences of the retrieved *B. rapa TIFY* genes were obtained from the *Brassica* database (http://brassicadb.org/brad/; Cheng et al., 2011). “TIFY” domains were confirmed using the web tool “SMART” from EMBL (http://smart.embl.de/smart/set_mode.cgi?GENOMIC=1) and by using the Basic Local Alignment Search Tool (BLAST) (http://www.ncbi.nlm.nih.gov/BLAST/) a protein homology study was conducted to confirm the putative *TIFY* genes in *B. rapa*.

### Sequence analysis of *B. rapa TIFY* genes

A thorough investigation on the primary structures (molecular weight and isoelectric point) of the *B. rapa* TIFY proteins was carried out using protParam (http://expasy.org/tools/protparam.html). “PIR,” an online web tool (http://pir.georgetown.edu/pirwww/search/multialn.shtml) was used to align the TIFY protein sequences. Number of introns and exons was predicted using the Gene Structure Display Server (GSDS) web tool aligning the CDS with the genomic sequences (Guo et al., 2007). The subcellular location of TIFY proteins in *B. rapa* was determined using ProtComp 9.0 from Softberry (http://linux1.softberry.com/berry.phtml) and Blast2GO software (http://www.blast2go.de). For the analysis of the *B. rapa* TIFY protein motifs MEME software (Multiple Em for Motif Elicitation, V4.9.0) was employed (Bailey et al., 2006). A search on the MEME suit was executed to identify distinctive motifs with the following parameters: (1) width of optimum motif ≥6 and ≤ 200; (2) maximum number of motifs to identify = 10. Important protein motifs were drawn manually. Online tool STRING 10 (http://string-db.org/) was employed to predict the TIFY protein association and interactions in *B. rapa*.

### Phylogenetic analysis of *B. rapa* TIFY proteins

TIFY protein sequences from *B. rapa*, rice, and *Arabidopsis* were aligned using ClustalX (Thompson et al., 1997). A phylogenetic tree was generated with the help of MEGA6.06 software using the Neighbor-Joining (NJ) algorithm (Tamura et al., 2013). Significance of the nodes was analyzed through bootstrapping with 1000 replicates. Pairwise gap deletion mode was used to assume that the divergent domains might contribute to the topology of the NJ tree.

### Chromosomal locations and gene duplication of *BrTIFYs*

A BLAST search (http://www.ncbi.nlm.nih.gov/BLAST/) was employed using all the *BrTIFY* genes against each other and gene duplications were considered when both the similarity and query coverage percentage of the candidate genes were >80% (Kong et al., 2013). Chromosomal positions of the candidate *BrTIFY* genes along with their subgenome information were obtained from the *Brassica* database (http://brassicadb.org/brad/; Cheng et al., 2011). Gene positions in the chromosomes and duplication lines among genes on the chromosomes were drawn manually. We aligned the duplicated *BrTIFY* CDS sequences using ClustalW (http://www.genome.jp/tools/clustalw/) to predict the divergences period. We estimated the synonymous rate (*Ks*), non-synonymous rate (*Ka*), and evolutionary constriction (*Ka/Ks*) between the duplicated pairs of *B. rapa TIFY* genes using codeml in the PAML package (Goldman and Yang, 1994). The formula *T* = *Ks/2R* was employed to calculate the divergence time, where *T* refers to divergence time, *Ks* refers to the synonymous substitutions per site, and *R*, for the rate of divergence of plant's nuclear genes. *R*-value was considered as 1.5 × 10^−8^ synonymous substitutions per site per year in case of dicotyledonous plants (Koch et al., 2000).

### Collection and preparation of plant material

For plant samples, we cultivated *B. rapa* “SUN-3061” at the Department of Horticulture, Sunchon National University, Korea. Fresh roots, stems, leaves, and flower buds of mature plants were harvested for the organ study. Collected plant parts were frozen immediately in liquid nitrogen, and stored at −80°C for RNA isolation. Two inbred lines of *B. rapa* ssp. *pekinensis*, Chiifu and Kenshin having two contrasting geographic origins were used for the cold stress treatment at 4°C. Chiifu originated in temperate regions and Kenshin originated in subtropical and tropical regions (Lee et al., 2013). Plants were cultured in a semi-solid medium maintaining an aseptic condition for 2 weeks. To induce the cold stress, plant samples (Chiifu and Kenshin) were transferred to the incubator at 4°C. For rest of the abiotic and biotic stress treatments, only *B. rapa* Chiifu was used. To impose the drought/desiccation stress plants were kept on the Whatmann 3 mm filter sheets. For salt, abscisic acid (ABA), jasmonic acid (JA), and salicylic acid (SA) treatments, plants were shifted to rectangular petri dishes (72 × 72 × 100 mm) containing 200 mM NaCl, 100 μM ABA, JA, and SA, respectively (Yanhui et al., 2006; Saha et al., 2015). Sampling of all the abiotic stress treated plants were done at 0, 1, 4, 12, 24, and 48 h.

For biotic stress treatment, a fungal inoculation by *Fusarium oxysporum* f.sp. *conglutinans* in *B. rapa* Chiifu were done according to Ahmed et al. (2013). Fungus- and mock-treated plants were sampled at 0, 3, 6 h, 1, 3, 6, 9, and 12 d. Sampling were done from the local (4th) and systemic (5th) leaves. Inoculations were done three times, and infection was verified by monitoring disease lesions on plant leaves. Stress treated leaf samples were collected and frozen immediately in liquid nitrogen and stored at −80°C until RNA isolation.

### Microarray analysis of *BrTIFY* expression under low temperature treatment

Low temperature-treated microarray data for 36 *BrTIFY* genes were recovered from the data of Jung et al. (2014). For that data, two contrasting *B. rapa* inbred lines Chiifu and Kenshin were treated with different cold and freezing temperatures *viz*. 4, 0, −2, and −4°C for 2 h. The transcript abundance values of the 36 *BrTIFY* genes were collected and analyzed further to generate a heat map using Cluster 3.0 and tree view software (http://bonsai.hgc.jp/~mdehoon/software/cluster/software.htm) with the centroid clustering option.

### Transcript level analysis of *BrTIFY* genes

An AMV one-step RT-PCR kit (Takara, Japan) was used for RT-PCR analysis. *B. rapa* candidate *TIFY* gene specific primers were used for RT-PCR expression analysis (Supplementary Table 1). For internal control *Actin* primers from *B. rapa* (FJ969844) were used. For PCR analysis 50 ng cDNA from the plant and flower organs was used as templates in master mixes comprised of 20 pmol each primer, 150 μM each dNTP, 1.2 U Taq polymerase, 1x Taq polymerase buffer, and double-distilled H_2_O in a total volume of 20 μL in 0.5-mL PCR tubes. The samples were kept in a thermal cycler for PCR reaction to the following conditions: pre-denaturing at 94°C for 5 min, followed by 30 cycles of denaturation at 94°C for 30 s, annealing at 55°C for 30 s, and extension at 72°C for 45 s, with a final extension for 5 min at 72°C.

The real-time quantitative PCR was conducted using 1 μL cDNA in a 20-μL reaction volume employing iTaqTM SYBR® Green Super-mix with ROX (California, USA). List of specific primers used for real-time PCR has given in Supplementary Table 1. For real-time PCR analysis, prepared samples were subjected to the following conditions: 10 min at 95°C, followed by 40 cycles at 95°C for 20 s, 58°C for 20 s, and 72°C for 25 s. The fluorescence was determined observing the last step of each cycle, and three replications were used per sample. Light cycler® 96 SW 1.1 (Roche, Germany) was used for amplification detection and data analysis.

## Results

### Identification of TIFY family genes in the *B. rapa* genome

**Figure 1:**
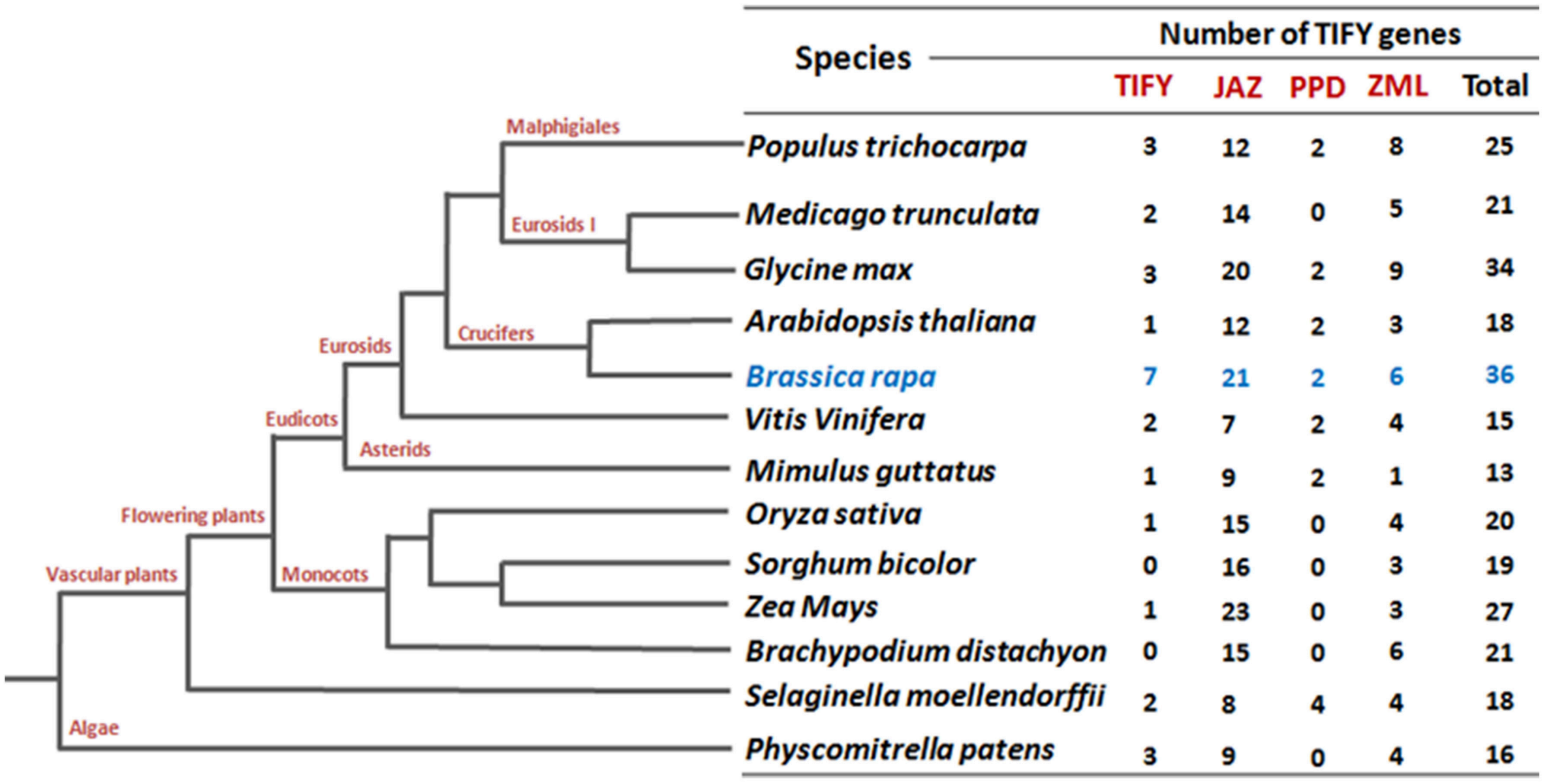
***TIFY* gene comparisons among different species**.

To identify all putative *TIFY* genes in *B. rapa*, we performed a search in the Swissprot annotations of the *Brassica* database (BRAD) using key word “TIFY.” (http://brassicadb.org/brad/). A set of 36 candidate *TIFY* genes was identified (Supplementary Table 2) and their protein and coding sequences (CDS) were retrieved (http://brassicadb.org/brad/). Besides, a blast search against characteristic TIFY domain sequences in the BRAD revealed 35 *B. rapa* TIFY proteins. We crosschecked all the TIFY protein sequences of both the sources and 36 *B. rapa* TIFY proteins were primarily predicted for further *in silico* analysis. A domain search using EMBL (http://smart.embl.de/smart/set_mode.cgi?GENOMIC=1) (“Pfam” web server option was also employed at the same time) with the corresponding *B. rapa* candidate TIFY protein sequences also confirmed 35 of them to contain the characteristic “TIFY” domain. The remaining candidate TIFY protein, which apparently lacks a “TIFY” domain, showed significant similarity to a TIFY protein of *Arabidopsis* (AT2G34600; JASMONATE-ZIM-DOMAIN PROTEIN 7) at the amino acid level (Supplementary Table 3). Notably, this number of *TIFY* genes in *B. rapa* (36) genome is the highest among all the crops have been reported to date (Vanholme et al., 2007; Ye et al., 2009; Zhang et al., 2012; Zhu et al., 2013; Li et al., 2015; Figure 1). The putative *TIFY* genes in *B. rapa* were named (*BrTIFY1a-BrTIFY11e*) based on the numbering system used in phylogenetic tree of *Arabidopsis* and rice (Supplementary Table 2).

### Sequence analysis of *BrTIFY* genes

**Figure 2:**
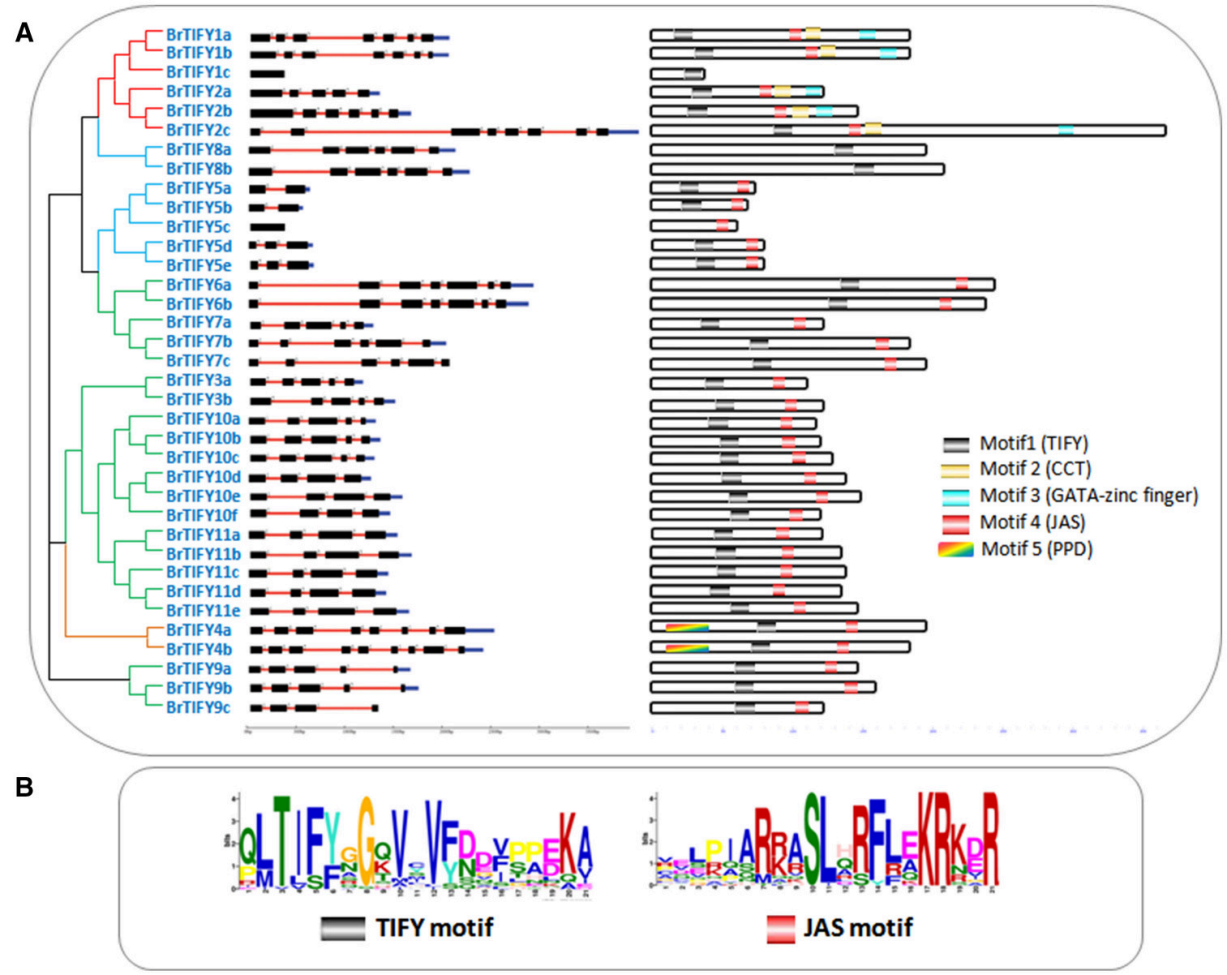
**(A)** Showing schematic representation of intron-exon distribution and motifs identified in *B. rapa* TIFY proteins. Intron–exon organization patterns of 36 *TIFY* genes are shown, along with their intron splicing patterns. On the right, different motifs (motif 1–5) are indicated by different colors, and the names of all members are shown on the left side of the figure, along with their phylogenetic relatedness, and **(B)** showing two motif logos (“TIFY” and “Jas”) with their amino acid conservation patterns.

The predicted sizes of the 36 *BrTIFYs* ranged from 112 to 363 amino acids (aa), and the predicted isoelectric point varied from 4.48 to 9.98 (Table 1). Further analysis of the protein domain organization showed that among 35 TIFY domain-containing proteins, 32 had both a TIFY domain and a Jas motif (Staswick, 2008) of SLX2FX2KRX2RX5PY. Only five proteins were found to bear a TIFY domain along with the CCT motif and C2C2-GATA zing finger domain that are characteristic of the ZML subfamily (Figure 2, Supplementary Figure 1). Two TIFY proteins containing the Jas motif were lacking the conserved proline (P) and tyrosine (Y) at the C-termini (Chung et al., 2009), and contained a PPD domain, which indicated that they were likely PPD subfamily proteins. Generally, a TIFY protein containing a TIFY domain along with a Jas motif has been considered as a member of the JAZ subfamily. Jas motifs are found to be located in the C-terminal region of the JAZ proteins which are essential for the interaction with LRR domain of COI1 (Katsir et al., 2008b). A highly conserved KR(K/N/R)(E/D)R sequence in the C-terminal end of the Jas motif has been observed which has been reported essential for nuclear localization signal of JAZ proteins (Withers et al., 2012; Supplementary Figure 2). More specifically, we also found conserved arginine (R) within the Jas motif (Figure 2), which is important for JAZ nuclear localization and interaction with MYC2 (Withers et al., 2012). Besides, seven proteins were found to contain the TIFY domain motif only. Accordingly, these seven proteins were supposed to be TIFY subfamily proteins.

**Table 1:** **Features of 36 TIFY proteins in ***B. rapa*****.

**Sl**.	**Protein name**	**Locus name**	**Protein**			**TIFY subfamily name**	**Nuclear location**
			**Length (aa)**	**MW (D)**	**P^I^**		
1.	BrTIFY1a	Bra013805	269	29,286.7	6.71	ZML	N
2.	BrTIFY1b	Bra019227	261	28,333.4	5.97	ZML	N
3.	BrTIFY1c	Bra031379	112	12,078.2	4.48	ZML	N
4.	BrTIFY2a	Bra014292	260	28,265.4	6.67	ZML	N
5.	BrTIFY2b	Bra031258	321	35,391.5	6.46	ZML	N
6.	BrTIFY2c	Bra023900	363	40,663.8	8.73	ZML	N
7.	BrTIFY3a	Bra002338	201	20,955.4	6.91	JAZ	N
8.	BrTIFY3b	Bra020135	221	23,108.9	4.97	JAZ	N
9.	BrTIFY4a	Bra036885	319	35,112.4	8.78	PPD	N
10.	BrTIFY4b	Bra039720	324	35,794.1	9.53	PPD	N
11.	BrTIFY5a	Bra005407	116	13,144.1	9.64	TIFY	N
12.	BrTIFY5b	Bra021923	117	13,444.4	9.89	TIFY	N
13.	BrTIFY5c	Bra022981	113	13,013.7	8.74	TIFY	N
14.	BrTIFY5d	Bra010794	130	14,971.7	9.85	TIFY	N
15.	BrTIFY5e	Bra032362	133	15,329.2	9.62	TIFY	N
16.	BrTIFY6a	Bra021281	353	37,579	9.3	JAZ	N
17.	BrTIFY6b	Bra022254	335	35,868.1	9.51	JAZ	N
18.	BrTIFY7a	Bra003947	220	24,348.5	8.82	JAZ	N
19.	BrTIFY7b	Bra007937	250	26,862.3	9.92	JAZ	N
20.	BrTIFY7c	Bra016193	249	26,979.5	9.85	JAZ	N
21.	BrTIFY8a	Bra011357	344	37,392.4	9.69	TIFY	N
22.	BrTIFY8b	Bra040086	357	38,775.8	9	TIFY	N
23.	BrTIFY9a	Bra006190	195	21,818.1	9.91	JAZ	N
24.	BrTIFY9b	Bra008846	196	21,819.2	9.91	JAZ	N
25.	BrTIFY9c	Bra023399	184	20,606.7	9.99	JAZ	N
26.	BrTIFY10a	Bra003778	222	24,320.4	9.08	JAZ	N
27.	BrTIFY10b	Bra008172	240	26,197.5	9.21	JAZ	N
28.	BrTIFY10c	Bra015880	245	26,810.2	9.2	JAZ	N
29.	BrTIFY10d	Bra016520	259	28,310.1	9.49	JAZ	N
30.	BrTIFY10e	Bra031065	273	29,810.6	9.49	JAZ	N
31.	BrTIFY10f	Bra025713	254	27,300	9.85	JAZ	N
32.	BrTIFY11a	Bra008033	273	30,157.8	9.11	JAZ	N
33.	BrTIFY11b	Bra016056	274	30,596.3	9.08	JAZ	N
34.	BrTIFY11c	Bra016604	270	30,017.6	7.7	JAZ	N
35.	BrTIFY11d	Bra025977	256	28,753.3	9.28	JAZ	N
36.	BrTIFY11e	Bra030986	299	33,020.9	9.12	JAZ	N

*aa, amino acid; D, Dalton; MW, molecular weight; P^I^, iso-electric point; N, nuclear*.

**Figure 3:**
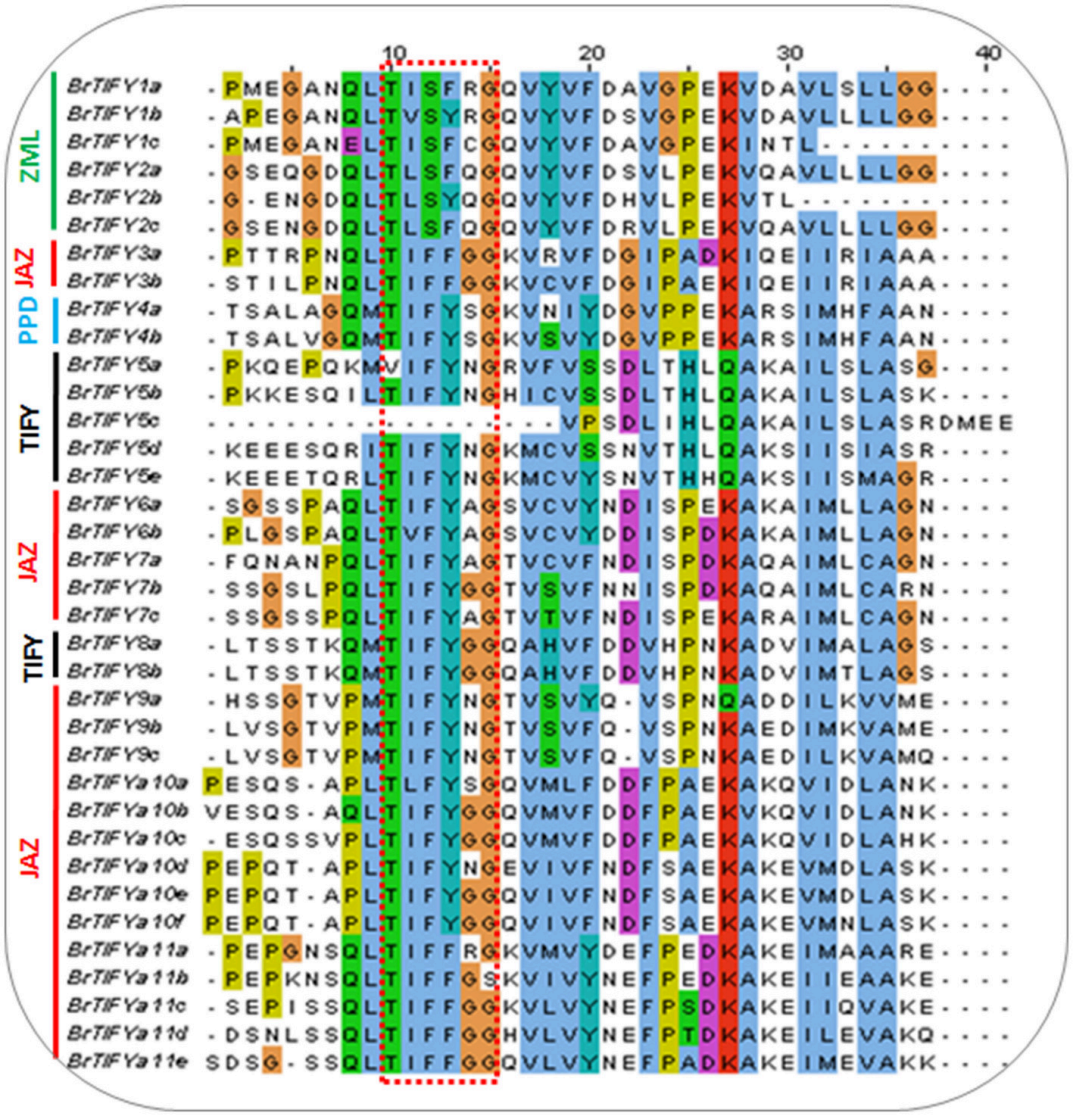
**TIFY domain alignment showing conserved “TIFY[X]G” motif among 36 ***B. rapa*** TIFY proteins**. Four subfamily proteins have been marked on the left of the figure.

To examine the variability among TIFY domains, we collected all the TIFY domain sequences and aligned them, revealing characteristic TIFY domains in different combinations such as TI[F/S]YXG, T[L/I]S[F/Y]XG, TVSYXG, VIFYXG, and TIFF[G/R]G (Figure 3). Specifically, all the TIFY (except BrTIFY5c) and PPD subfamily proteins and 14 out of 21 JAZ proteins shared a common sequence “TIFYXG” in the TIFY domain. Notably, all the phenylalanine (F) positions of the “TIFY” motif in the ZML proteins were replaced by serine (S). In addition, they showed inconsistent “TIFY” motif conservation as T[L/I]S[F/Y]XG in their domain sequences.

The DNA sequences of the 36 *BrTIFY* genes were determined based on the *B. rapa* whole-genome sequence. Exon-intron analysis of 36 *BrTIFY* genes revealed that most of the genes contained more than one exon (up to 9) with fairly consistent splicing patterns within different groups (Figure 2). Specifically, genes from the ZML subfamily bore 7–8 exons with the splicing pattern “0/2.” Two PPD subfamily genes possessed the most exons (8–9) and had “1/0/2” splicing patterns. The JAZ subfamily members were identified to have 4–5 exons with a splicing pattern “1/2” in most cases. Finally, the TIFY subfamily genes exhibited the highest variability; they contained 1 to 6 exons with splicing pattern “0/1/2.” We have studied the subcellular localization of the 36 BrTIFY proteins using Protcomp 9.0 from Softberry and Blast2GO software. Consistent with being transcription factor family proteins, all the BrTIFYs were predicted to be localized in the nucleus (Table 1).

### Phylogenetic analysis of *B. rapa* TIFY proteins

**Figure 4:**
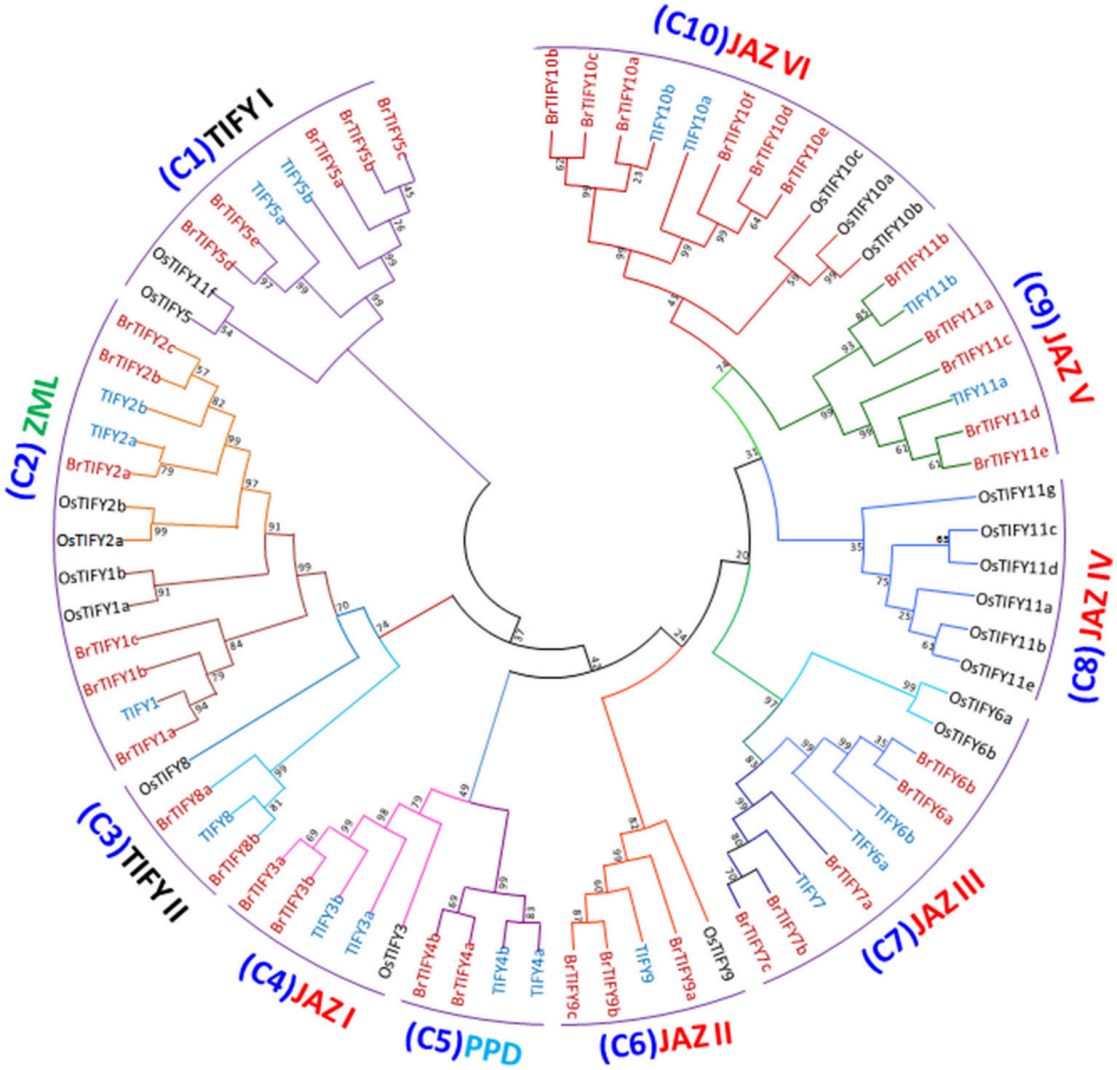
**Phylogenetic relatedness and subgroup designations of TIFY proteins from ***B. rapa***, ***Arabidopsis*** and rice**. Maximum-likelihood tree showing the relationships among 36 TIFY proteins from *B. rapa*, 18 from *Arabidopsis* and 20 from rice. The numbers on the branches indicate bootstrap support values from 1000 replications.

A phylogenetic tree was generated using the sequence alignments of the 36 full-length TIFY proteins to analyze the evolutionary relationships among the BrTIFY proteins and 18 TIFY proteins from *Arabidopsis* as well as 20 from rice. The 74 TIFY proteins were grouped into ten clades (C1-10) (Figure 4). Among the 10 clades, C1 and C3 were formed with the TIFY proteins containing only the TIFY domain, and these two clades were named as TIFY I and TIFY II. The TIFY I group included five proteins (BrTIFY5a, 5b, 5c, 5d, and 5e) and the TIFYII group had two proteins (BrTIFY8a and 8b). In C2, ZML proteins from *B. rapa, Arabidopsis* and rice were gathered together. Six BrTIFY proteins (BrTIFY1a, 1b, 1c, 2a, 2b, and 2c) were grouped with corresponding proteins from the *Arabidopsis* and rice ZML subfamily. Surprisingly, BrTIFY1c, which does not have a CCT or a GATA-Zn finger domain, was placed in the ZML subfamily. Significant sequence similarity outside of the CCT and GATA-Zn finger domain might explain its presence in this subfamily. Two PPD proteins (BrTIFY4a and 4b) from *B. rapa* formed C5. These two proteins contain partial Jas (lack conserved PY) motif and additionally possess a PPD domain in the N-terminal region. Due to Jas motifs in their C-terminal regions, these two proteins had close relationships with C4, which also contained proteins with Jas motifs and was designated as JAZ I. Notably, 21 BrTIFY JAZ proteins were found in six clades (C4, C6, C7, C8, C9, and C10, which we named as JAZ I, JAZ II, JAZ III, JAZ IV, JAZ V, and JAZ VI), indicating that there is a broader phylogenetic relationship within this subset of proteins. Specifically, 16 BrTIFY (BrTIFY3a, 3b, 6a, 6b, 7a, 7b, 7b, 9a, 9b, 9c, 10a, 10b, 10c, 10d, 10e, and 10f) proteins were grouped within four JAZ subgroups (JAZ I, JAZ II, JAZ III, and JAZ VI) with proteins from *Arabidopsis* and rice. The other 5 BrTIFY JAZ proteins (BrTIFY11a, 11b, 11c, 11d, and 11e) were grouped in C9 (JAZ V) with related proteins from *Arabidopsis*. A rice-specific JAZ group was formed with six OsTIFY JAZs in C8, which is designated as JAZ IV. It should be noted that in the phylogenetic tree, BrTIFY proteins were more commonly present with *Arabidopsis* TIFY proteins in a sister clade rather than forming clades with rice TIFY proteins. This signifies that the majority of *B. rapa* TIFY proteins are more closely related to those of *Arabidopsis* than those of rice, which is consistent with the fact that both *B. rapa* and *Arabidopsis* are eudicots and diverged more recently from a common ancestor than from the lineage that leads to monocots. Some interesting evolutionary relationships were observed from the above phylogenetic classification. For example, there was an approximately 1:3 ratio in the number of *Arabidopsis* TIFY homologs to those in *B. rapa*. Comparative genomic analysis has confirmed that *B. rapa* has experienced genome triplication since its divergence from *Arabidopsis* (Song et al., 2014).

### Chromosomal location of *TIFY* genes and their expansion patterns in *B. rapa*

**Figure 5:**
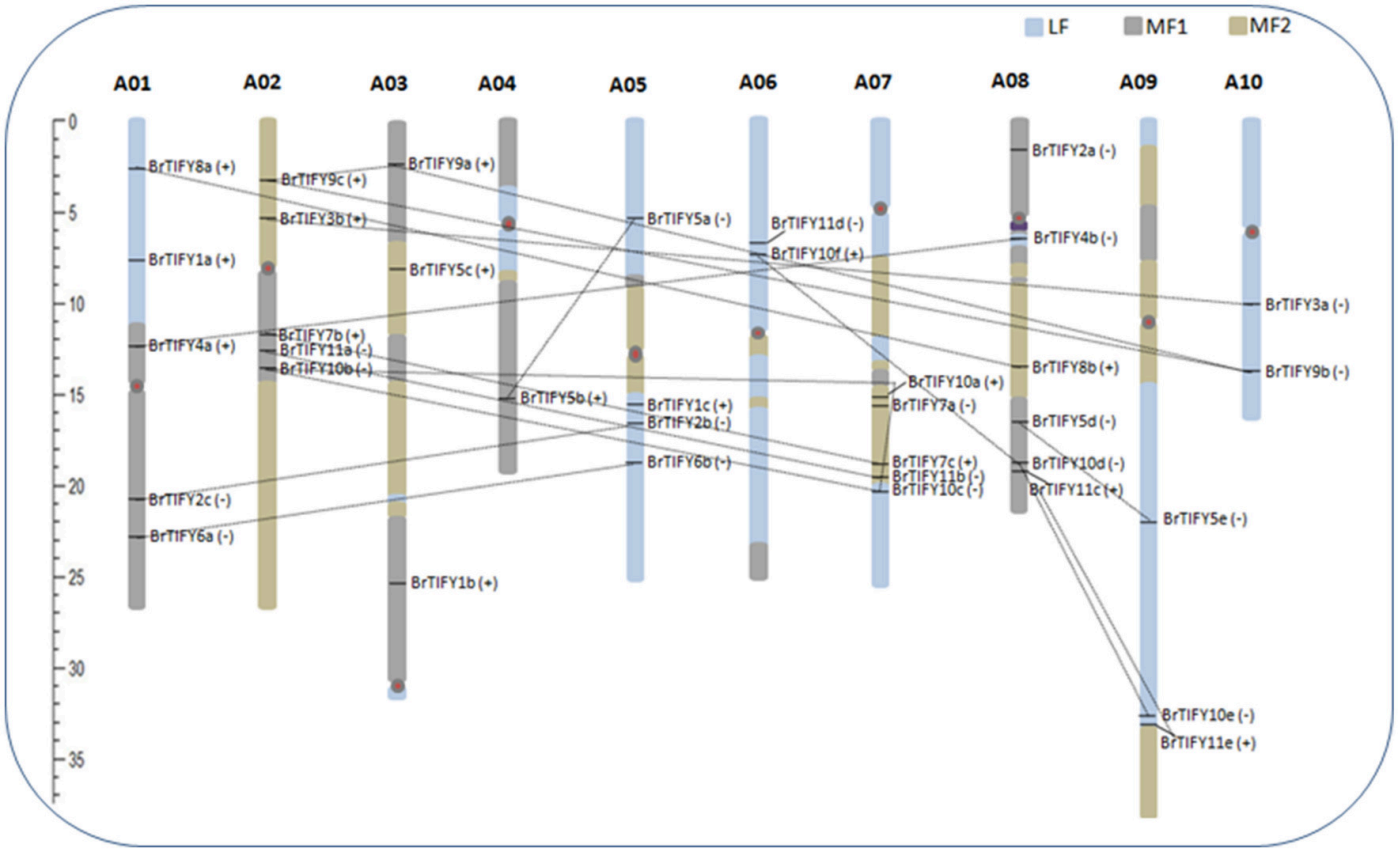
**Distribution of ***TIFY*** genes on the 10 chromosomes and three subgenomes of ***B. rapa*****. Different colored blocks on chromosomes are representing three subgenomes. *TIFY* genes are shown on the right of each chromosome. The positive (+) and negative (−) signs following each gene represent forward and reverse orientation of the respective gene, respectively, on the chromosome or subgenome. Genes are in duplicated segments of genome are joined by black dotted lines. Gene positions and chromosome size can be measured using the scale on the left of the figure.

We retrieved the physical locations of the 36 *TIFY* genes on the 10 chromosomes of *B. rapa* (Figure 5). The highest number of *TIFY* genes was found on chromosome 8 (6 genes). Chromosomes 1, 2, and 7 had 5 *TIFY* genes each and chromosome 4 contained only 1 *TIFY* gene. We also predicted the proportions of *BrTIFY* genes among three subgenomes of *B. rapa* (LF, MF1, and MF2). The most *BrTIFY* genes (15) were found in the LF subgenome, whereas 13 and 8 genes were found in the MF1 and MF2 subgenomes, respectively. Duplication analysis revealed that 22 out of 36 *BrTIFY* genes (61.1%) were present in two or more copies. This gene duplication likely occurred as a result of whole genome duplication (WGD) or segmental duplications (black dotted lines in Figure 5); no evidence of tandem duplication was observed in any of the chromosomes. Higher frequencies of segmental duplications generated many homologs of *TIFY* genes along all chromosomes of *B. rapa* (black dotted lines in Figure 5). Moreover, we estimated the *Ka/Ks* ratios as less than one (1) for all the duplicated *TIFY* gene pairs in *B. rapa* genome which indicate about their purifying selections. The divergence time for duplicated *BrTIFY* genes revealed that it took 2.5–13.05 MYA (Table 2). The divergence period of six *TIFY* genes (*BrTIFY6a-BrTIFY6b, BrTIFY10a-BrTIFY10b, BrTIFY10a-BrTIFY10c, BrTIFY10b-BrTIFY10c, BrTIFY11a-BrTIFY11b*, and *BrTIFY11c-BrTIFY11e*) ranged from 5.2 to 7.6 MYA, indicating that divergence of these genes took place during the *Brassica* triplication events (5~9 MYA) (Woodhouse et al., 2014). Five gene pairs (*BrTIFY3a-BrTIFY3b, BrTIFY4a-BrTIFY4b, BrTIFY5a-BrTIFY5b, BrTIFY5d-BrTIFY5e*, and *BrTIFY7b-BrTIFY7c*) ranged their divergence time from 11.14 to 13.05 MYA, which indicate that these duplication events occurred during the divergence of *B. rapa* from *Arabidopsis* (9.6–16.1 MYA) (Wang et al., 2011). Finally, seven pairs of duplicated *BrTIFY* genes (*BrTIFY2b-BrTIFY2c, BrTIFY8a-BrTIFY8b, BrTIFY9a-BrTIFY9b, BrTIFY9a-BrTIFY9c, BrTIFY9b-BrTIFY9c, BrTIFY10d-BrTIFY10e*, and *BrTIFY10d-BrTIFY10f*) were identified as they diverged recently (2.5–4.22 MYA).

**Table 2:** **Estimated ***Ka/Ks*** ratios of the duplicated ***TIFY*** genes with their divergence time in ***B. rapa*****.

**Duplicated gene pairs**	***Ka***	***Ks***	***Ka/Ks***	**Type of duplication**	**Purifying selection**	**Time (MYA)**
*BrTIFY2b-BrTIFY2c*	0.2985	0.1267	0.4244	WGD/segmental	Yes	4.22
*BrTIFY3a-BrTIFY3b*	0.1370	0.3682	0.3720	WGD/segmental	Yes	12.27
*BrTIFY4a-BrTIFY4b*	0.1203	0.3917	0.3072	WGD/segmental	Yes	13.05
*BrTIFY5a-BrTIFY5b*	0.1413	0.3750	0.3767	WGD/segmental	Yes	12.5
*BrTIFY5d-BrTIFY5e*	0.0748	0.3636	0.2056	WGD/segmental	Yes	12.12
*BrTIFY6a-BrTIFY6b*	0.0883	0.2297	0.3843	WGD/segmental	Yes	7.6
*BrTIFY7b-BrTIFY7c*	0.1552	0.3341	0.4646	WGD/segmental	Yes	11.14
*BrTIFY8a-BrTIFY8b*	0.3280	0.1149	0.3502	WGD/segmental	Yes	3.8
*BrTIFY9a-BrTIFY9b*	0.2670	0.1181	0.4424	WGD/segmental	Yes	3.94
*BrTIFY9a-BrTIFY9c*	0.2664	0.1243	0.4665	WGD/segmental	Yes	4.14
*BrTIFY9b-BrTIFY9c*	0.4229	0.0751	0.1776	WGD/segmental	Yes	2.5
*BrTIFY10a-BrTIFY10b*	0.3287	0.1687	0.5132	WGD/segmental	Yes	5.6
*BrTIFY10a-BrTIFY10c*	0.3808	0.1834	0.4816	WGD/segmental	Yes	6.1
*BrTIFY10b-BrTIFY10c*	0.4192	0.1560	0.3722	WGD/segmental	Yes	5.2
*BrTIFY10d-BrTIFY10e*	0.3216	0.0995	0.3092	WGD/segmental	Yes	3.31
*BrTIFY10d-BrTIFY10f*	0.2887	0.0951	0.3295	WGD/segmental	Yes	3.17
*BrTIFY11a-BrTIFY11b*	0.4500	0.1840	0.4088	WGD/segmental	Yes	6.13
*BrTIFY11c-BrTIFY11e*	0.3774	0.1771	0.4692	WGD/segmental	Yes	5.9

*Ka, non-synonymous substitution rate; Ks, synonymous substitution rate; MYA, Million years ago*.

### Expression patterns of *TIFY* genes in *B. rapa*

#### Expression profiles of *BrTIFY JAZ* genes in various organs

**Figure 6:**
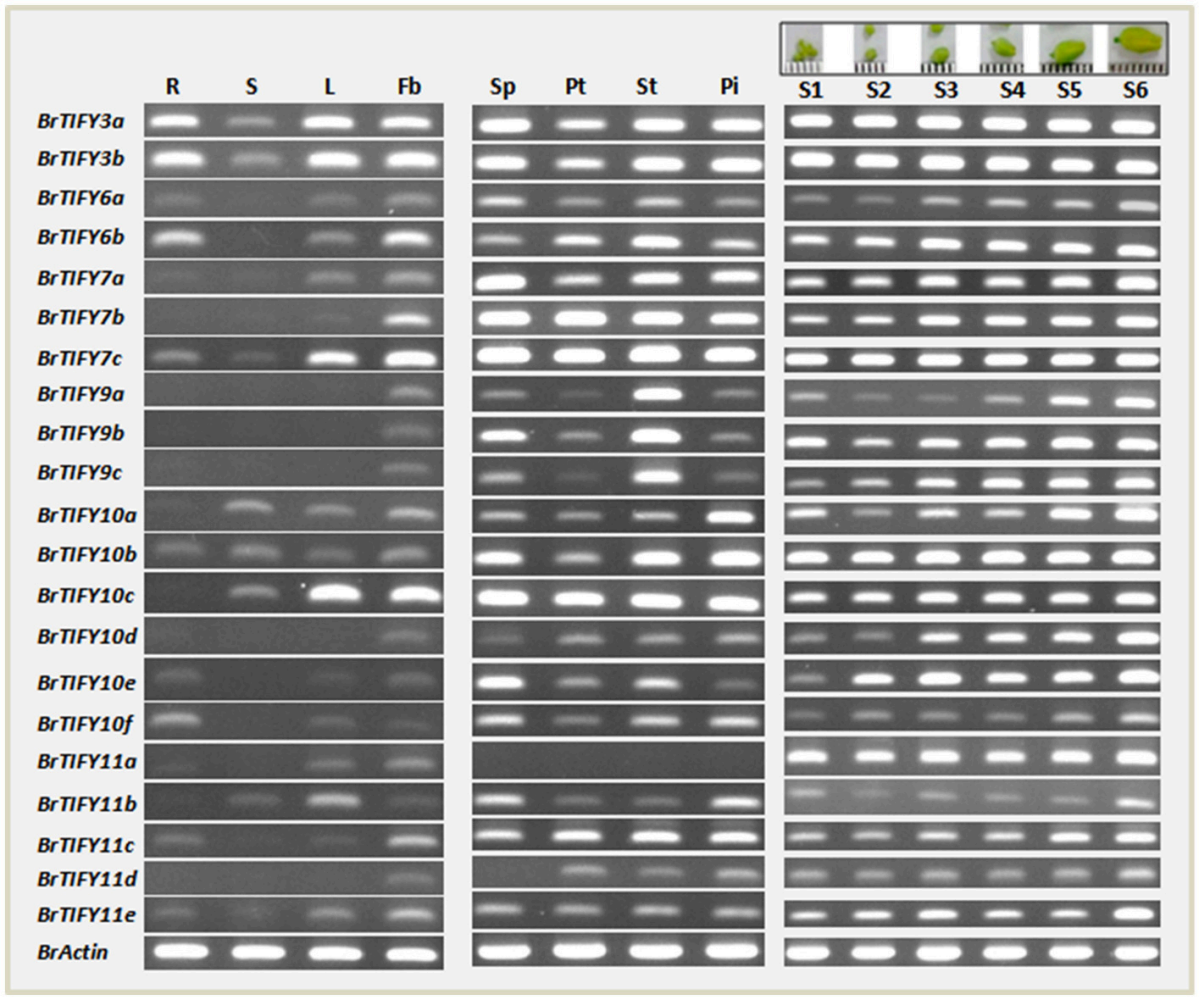
**RT-PCR expression of 21 JAZ subfamily genes in different tissues of ***B. rapa*****. Data from lane 1–14 showing expression in roots (R), stems (S), leaves (L), flower buds (Fb), sepal (SP), petal (Pt), stamen (St), pistil (Pi), and six flower growth stages (young to mature buds are marked S1–S6 at the top of the figure) of *B. rapa*. Name of genes are indicated on the left of the figure.

To gain insight into the potential developmental roles of *B. rapa TIFY* genes, we selected the *JAZ* genes for further analysis, as these genes make up the largest portion of the *TIFY* genes and have been reported to be involved in growth regulatory functions related to organ, especially flower development (Chini et al., 2007; Chung et al., 2009; Song et al., 2011; Hori et al., 2014; Yuan and Zhang, 2015). First, we conducted semi-quantitative RT-PCR analysis using specific primers with equal amounts of cDNA templates prepared from the mRNA of roots, stems, leaves, and flower buds of healthy, unstressed *B. rapa* plants to study the organ-specific expression patterns of *JAZ* genes. The genes showed differential expression patterns, with only four genes (*BrTIFY3a, 3b, 7c*, and *10b*) expressed in all four tested organs (Figure 6). All 21 JAZ subfamily genes were predominantly expressed in the flower buds of *B. rapa*, which indicates that genes from this subfamily might have important roles related to growth and development of *B. rapa* flowers.

Subsequently, we investigated the expression of the *BrTIFY JAZ* genes in four floral tissues *viz*. sepal, petal, stamen, and pistil of *B. rapa* flowers. Other than *BrTIFY11a*, which had no expression in any of the floral tissues, all of these genes exhibited widespread expression in most of the floral organs. The omnipresent expression of these genes in the floral tissues of *B. rapa* further indicates that these genes might be important factors for flower development in *B. rapa*. For further verification, we observed the expression patterns of all 21 *JAZ* genes throughout the development of *B. rapa* flowers (young to mature bud stages; S1–S6; Figure 6). We found all of these genes to be expressed in different growth stages of flower buds in two patterns. Four genes (*BrTIFY3a, 3b, 7c*, and *10b*) expressed ubiquitously with equal transcript abundance levels from young to the mature bud stage. Surprisingly, *BrTIFY11a* also showed widespread expression in all the flower bud development stages. Besides, we observed that the rest of the *JAZ* genes (16 genes) showed gradual increments of their transcript levels from young to the mature bud stage. These genes might play greater roles in the mature buds than in the young buds.

#### Microarray expression analysis of *BrTIFY* genes in response to cold and freezing treatments

**Figure 7:**
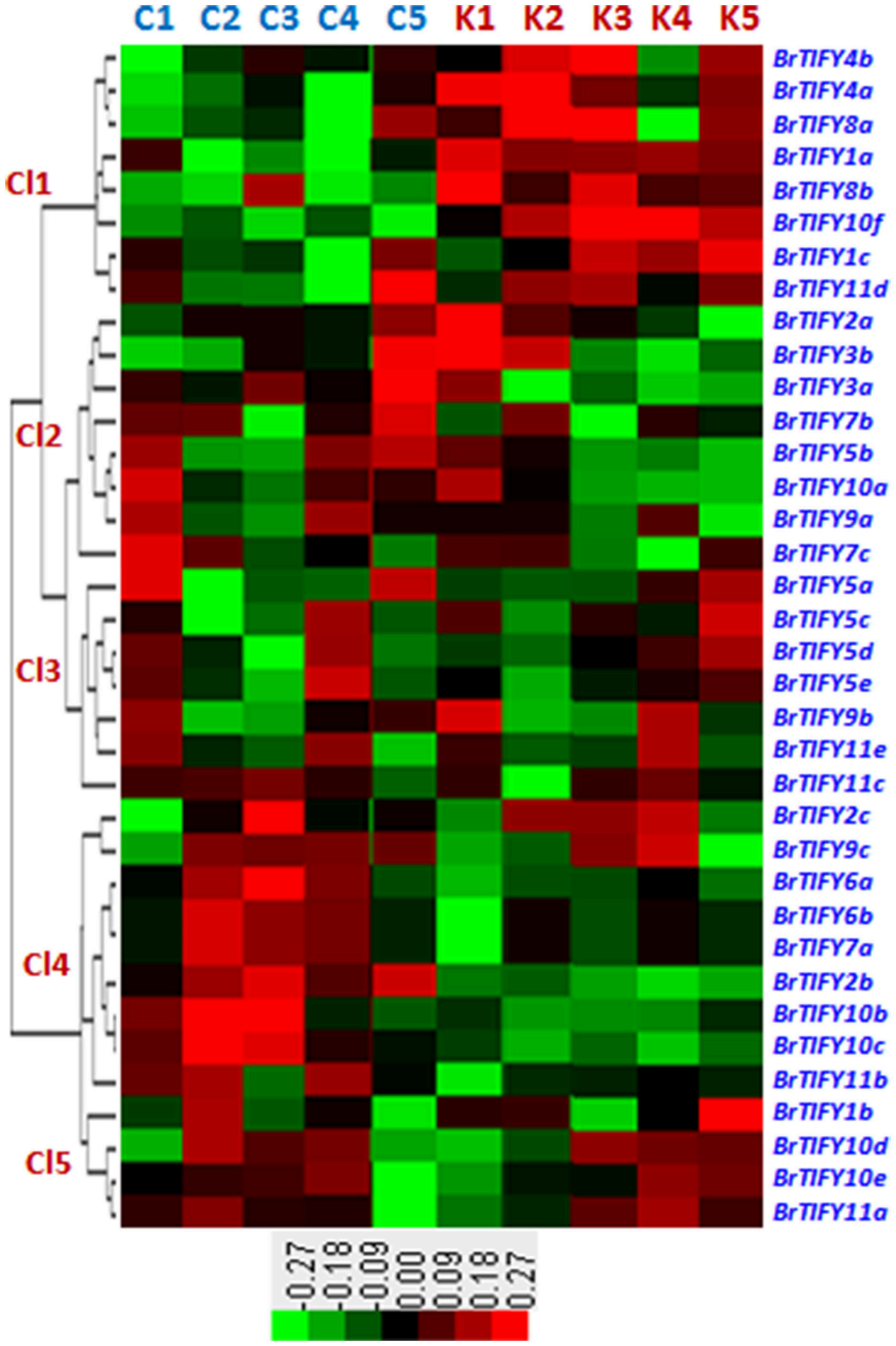
**Microarray expression represented as heat map and hierarchical clustering of ***36 TIFY*** genes against different low temperatures such as control (C1 and K1), 4°C (C2 and K2), 0°C (C3 and K3), −2°C (C4 and K4), and −4°C (C5 and K5) in ***B. rapa*****. Here, “C” and “K” stand for “Chiifu” and “Kenshin” two inbred lines of *B. rapa*, respectively. Expression clusters are shown in the left (Cl1–Cl5) and gene name against each expression has mentioned at right side. Color bars with values at the base represent differential expression in microarray.

We studied microarray expression data of the 36 *TIFY* genes using a previously published data set, wherein two contrasting inbred lines of *B. rapa*, Chiifu and Kenshin, were treated with cold and freezing stresses (4, 0, −2, and −4°C) for 2 h (Jung et al., 2014; Figure 7). Chiifu and Kenshin have contrasting geographic origins; Chiifu originated in temperate regions and Kenshin originated in subtropical and tropical regions. Hence, they are expected to respond differently to cold and freezing temperatures. In fact, Kenshin experiences severe damage upon exposure to low temperatures, while Chiifu does not exhibit such damage (Dong et al., 2014). To investigate expression patterns, we constructed a heat map using the differential transcript values for the *TIFY* genes in response to cold and freezing treatments in the two contrasting inbred lines of *B. rapa*. We divided the expression patterns of the 36 *TIFY* genes into five clusters (Cl1 to Cl5). In Cl1, eight *TIFY* genes [two *PPD* (*BrTIFY4a, 4b*), two *TIFY* (*BrTIFY8a, 8b*), two *ZML* (*BrTIFY1a, 1c*), and two *JAZ* (*BrTIFY10f, 11d*)] showed higher expression in response to both cold and freezing temperature in Kenshin than in Chiifu. The eight *TIFY* genes (six *JAZ*, one *TIFY* and one *ZML* genes) in Cl2 showed higher expression during the normal temperature (22°C) in both lines. Notably, three Chiifu *JAZ* genes (*BrTIFY3a, 3b*, and *7b*) in this cluster showed the highest expression in response to freezing temperature (−4°C). In Cl3, seven genes formed a group, four of which were from TIFY (*BrTIFY5a, 5c, 5d*, and *5e*) and the other three (*BrTIFY9b, 11c*, and *11e*) were from JAZ subfamily. Generally, genes of this cluster showed very low expression upon cold and freezing in the two lines. In Cl4, nine *TIFY* genes in *B. rapa* Chiifu exhibited the highest transcript abundance in response to cold and freezing temperatures. Surprisingly, among these nine genes, seven were from the JAZ subfamily (*BrTIFY6a, 6b, 7a, 9c, 10b, 10c*, and *11b*). Finally, in Cl5, four *TIFY* genes (three *JAZ*; *BrIFY11a, 11d, 11e*, and one *ZML*; *BrTIFY1b*) showed higher transcript abundance upon cold and freezing in both of the *B. rapa* lines. As a whole, we found that most of the *JAZ* genes in Chiifu were more highly induced by cold and freezing than those in Kenshin. Thus, we speculate that the set of *TIFY* genes exhibiting higher expression in Chiifu upon cold and freezing treatments might play roles in the cold and freezing tolerance of *B. rapa* Chiifu. Conversely, the *TIFY* genes that showed higher expression in Kenshin upon cold and freezing temperature might be factors that make Kenshin cold and freezing susceptible.

#### Expression analysis of *JAZ* genes under abiotic stress conditions in *B. rapa*

**Figure 8:**
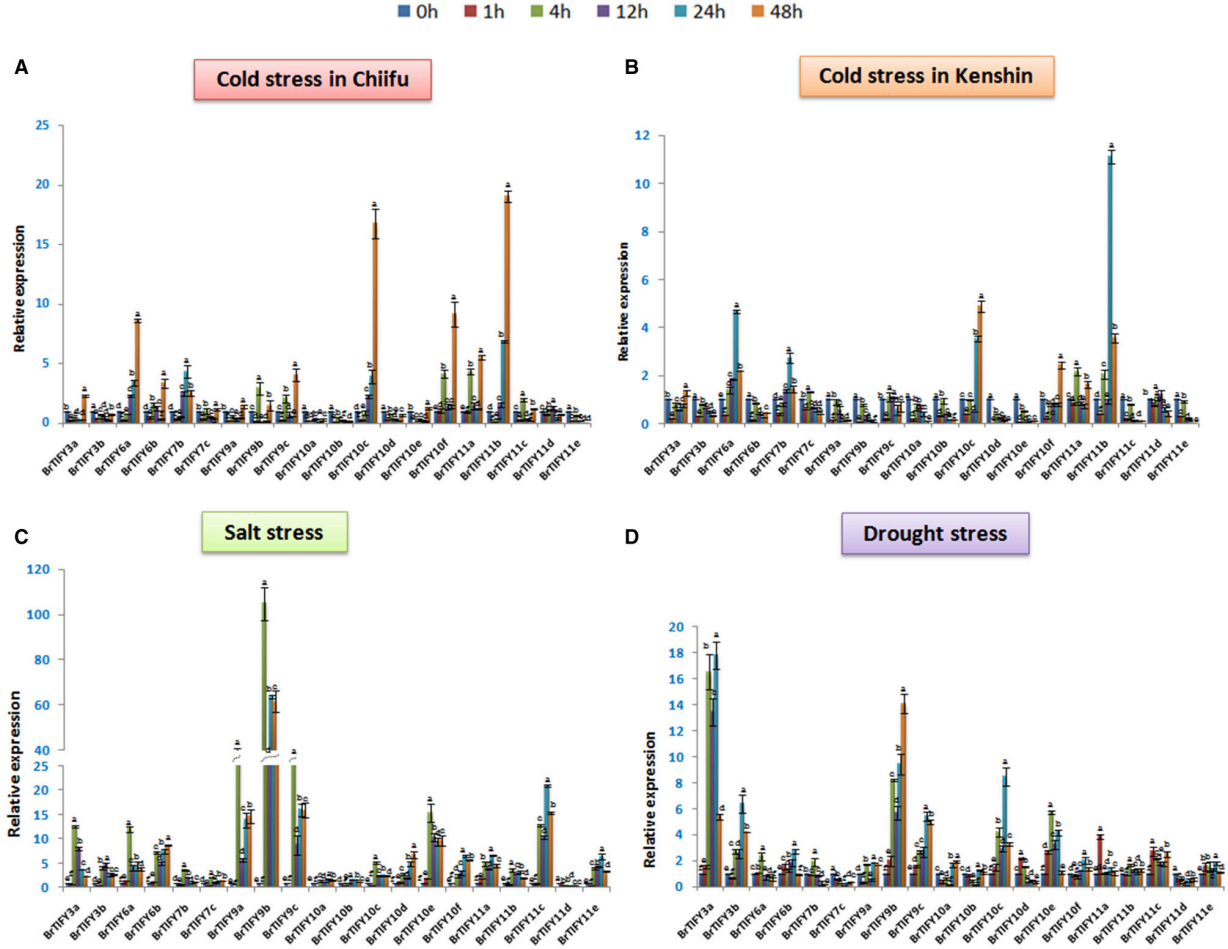
**Showing RT-qPCR expression analyses of 20 ***B. rapa JAZ*** genes in response to (A,B) cold, (C) salt, and (D) drought stresses (0–48 h)**. The error bars represent the standard error of the means of three independent replicates. Values denoted by the same letter did not differ significantly at *P* < 0.05 according to Duncan's multiple range tests.

Different abiotic stresses such as low temperature, drought and salinity adversely affect plant growth and development. In the microarray expression data, we found that most of the *TIFY* genes, specifically the *JAZ* genes showed comparatively higher expression under low temperature conditions. Therefore, to further elucidate and to validate the responses of the *JAZ* genes to cold stress we used two inbred lines of *B. rapa*, Chiifu and Kenshin for qPCR expression analysis (Figures 8A,B). Additionally, we examined responses of these genes against salt and drought treatment (Figures 8C,D). We studied all the expressions on a time course basis (0, 1, 4, 12, 24, and 48 h).

In Chiifu, most of the *JAZ* genes were up-regulated in response to cold treatment. For example, *BrTIFY3a, 6a, 6b, 7b, 9b, 9c, 10c, 10f, 11a, 11b*, and *11c* exhibited several fold up-regulation compared to the control under cold treatment. The highest up-regulation was shown by *BrTIFY11b* (>20-fold). *BrTIFY6a* and *10c* showed gradually increasing expression from 0 to 48 h, when they up-regulated by more than 8- and 15-fold, respectively. *BrTIFY3a, 6a, 6b, 7b, 7c, 9a, 9b, 9c, 10c, 10e, 11a, 11b, 11c*, and *11d* showed down-regulation at 1 h just after being induced by cold treatment. After that, from 4 to 48 h they showed differential up-and down-regulation during the cold period. In comparison with the microarray data, we also found that these genes also had differential expression against the cold and freezing temperatures. In general, the up-regulated *BrTIFY JAZ* genes showed their highest transcript levels at 48 h and exhibited differential expression patterns at other time points. We also found down-regulation of *BrTIFY3b, 10a, 10b, 10d*, and *11e* throughout the cold treatment period and their down-regulation might be related with the cold resistance of Chiifu (Figure 8A). By contrast, in Kenshin, most of the *JAZ* genes were actually down-regulated or up-regulated to lower levels in response to cold stress. For example, *BrTIFY 9b* and *9c* in Kenshin were down-regulated gradually from 0 to 48 h during cold stress. The *JAZ* genes highly up-regulated in Chiifu (*BrTIFY 6a, 10c*, and *11b*) also showed up-regulation in Kenshin, but their levels of up-regulation were only about half as high (Figure 8B). These results suggest that the highly up-regulated *JAZ* genes in Chiifu might be related to the cold resistance of this line and, by contrast, the very low activity or no activity of these genes in response to cold stress in Kenshin might be responsible for the cold susceptibility of this line at differential levels.

Under salt stress in Chiifu, we identified some *JAZ* candidates that were up-regulated. In general, most of the Chiifu *JAZ* genes were highly induced at 4 h of treatment. And, at 1 h, most of these genes were induced only at low levels or were down-regulated. The highest up-regulation was exhibited by *BrTIFY9b*, which showed >100-fold up-regulation at 4 h. Two other members of the JAZ II (*BrTIFY9a* and *9c*) also showed up-regulation, by >40- and >25-fold, respectively, compared to the reference. At the same time, other members of JAZ subfamily *viz. BrTIFY3a, 3b, 6a, 6b, 10d, 10e, 10f, 11c*, and *11e* showed considerable and differential response to salt stress*. BrTIFY3a, 6a, 7b, 7c, 9a, 9b, 9c, 10c, 10e*, and *11b* showed their highest up-regulation at 4 h which has been down-regulated a bit afterwards and remained static till 48 h. Besides, *BrTIFY6b, 10d, 10f, 11a, 11c*, and *11e* were highly induced at 24/48 h of salt treatment. We found only *BrTIFY11d* to be down-regulated completely after salt treatment (Figure 8C).

In the case of exposure to drought stress, *JAZ* genes in Chiifu showed variable transcript abundance at different time points. Notably, 18 *JAZ* genes out of 20 were up-regulated in response to drought stress. Most of the genes started to up-regulate differentially from 4 h to onward. The highest induction was recorded for *BrTIFY3a*, which showed >15-fold up-regulation compared to the control. *BrTIFY9b, 9c, 10c*, and *10e* also exhibited notable up-regulation in response to drought stress. Among the drought responsive *JAZ* genes *BrTIFY3a, 3b, 6b, 9a, 9b, 9c, 10a, 10b, 10c, 10f*, and *11e* showed highest up-regulation during late hours of treatment that is from 24 to 48 h. Only two *JAZ* genes, *viz. BrTIFY7c* and *11d*, showed complete down-regulation in response to drought treatment (Figure 8D).

It was interesting that most of the *JAZ* genes first showed up-regulation during early hours of salt and drought treatment followed by slight down regulation and then again up-regulated. The *JAZ* genes highly induced in response to salt and drought treatments might be good candidates for developing resistance against those stresses in *B. rapa*.

#### Expression patterns of *JAZ* genes in response to hormone treatments in *B. rapa*

**Figure 9:**
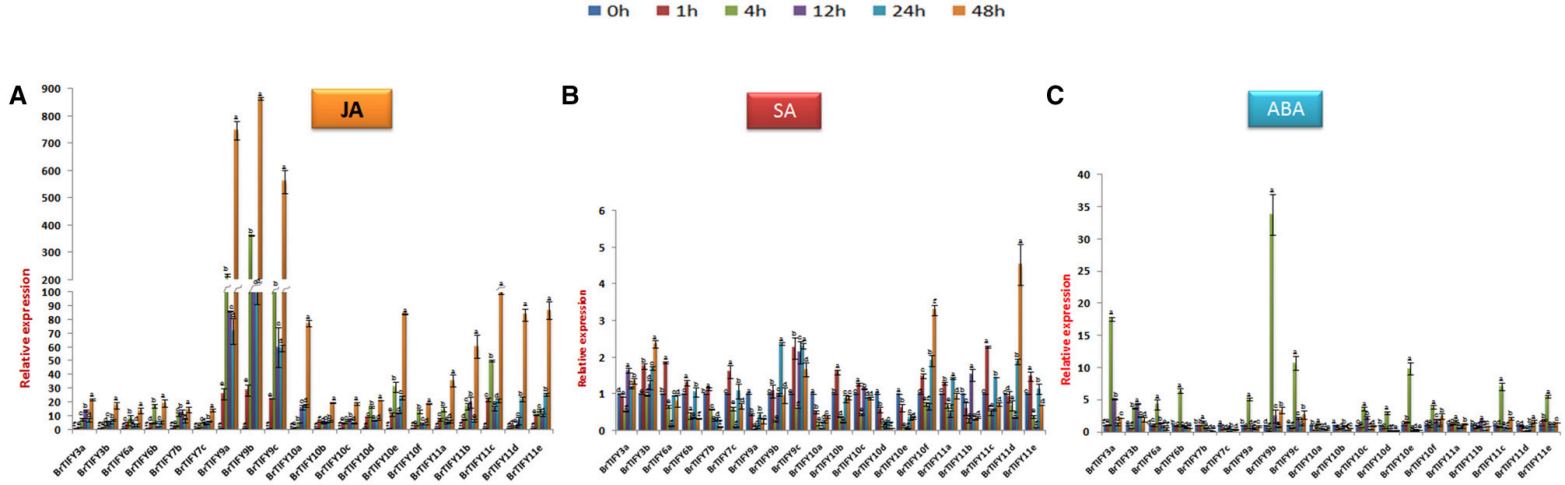
**Showing RT-qPCR expression analyses of 20 ***B. rapa JAZ*** genes in response to (A) JA, (B) SA, and (C) ABA treatment (0–48 h)**. The error bars represent the standard error of the means of three independent replicates. Values denoted by the same letter did not differ significantly at *P* < 0.05 according to Duncan's multiple range tests.

Phytohormones play important roles allowing plants to respond against stresses (Fujita et al., 2006; Huang et al., 2008). JAZ subfamily genes have been demonstrated to function in the JA signaling pathway in *Arabidopsis* (Chini et al., 2007; Thines et al., 2007). Here, we studied the responses of *JAZ* genes to JA, ABA, and SA in *B. rapa* Chiifu. Following treatment with JA, all *JAZ* genes in *B. rapa* were induced highly, where the highest up-regulation was recorded for *BrTIFY9b* (>800-fold), while *9a* and *9c* showed >700 and >500-fold up-regulation, respectively. The other *JAZ* genes had relative transcript abundance from >20- to 100-fold up-regulation at 48 h. Over the rest of the time course, they showed variable expression. Notably, all the genes showed the highest up-regulation at 48 h (Figure 9A). In response to SA treatment, most of the *JAZ* genes were induced moderately, with the highest up-regulation shown for *BrTIFY11d* (>5-fold). Conversely, *BrTIFY9a, 10a, 10d*, and *10e* were down-regulated completely after exogenous SA treatment. The rest of the genes showed up-regulation at least at one time point in response to SA, where the maximum fold change with respect to the control was >3 (Figure 9B). After exogenous ABA treatment in Chiifu, *JAZ* genes were expressed differentially. *BrTIFY3a, 3b, 6a, 6b, 7b, 9a, 9b, 9c, 10a, 10c, 10d, 10e, 10f, 11a, 11c*, and *11e* showed drastic up-regulation and the highest induction for each gene was recorded at 4 h. Their expression during other time points (1, 12, 24, and 48 h) were very low. Notably, *BrTIFY6a, 6b, 7b, 10a, 10d, 10e*, and *11b* were found to be down-regulated below the reference (0 h) level. The expression of *BrTIFY9b* was consistent and showed the highest up-regulation (>35-fold) upon ABA treatment among all of the *JAZ* genes. Two other copies of this gene (*9a* and *9c*) also showed up-regulation after ABA treatment. Besides, only two genes *BrTIFY7c* and *10b* were down-regulated completely after ABA treatment (Figure 9C).

#### Expression patterns of *JAZ* genes upon *Fusarium* infection in *B. rapa*

**Figure 10:**
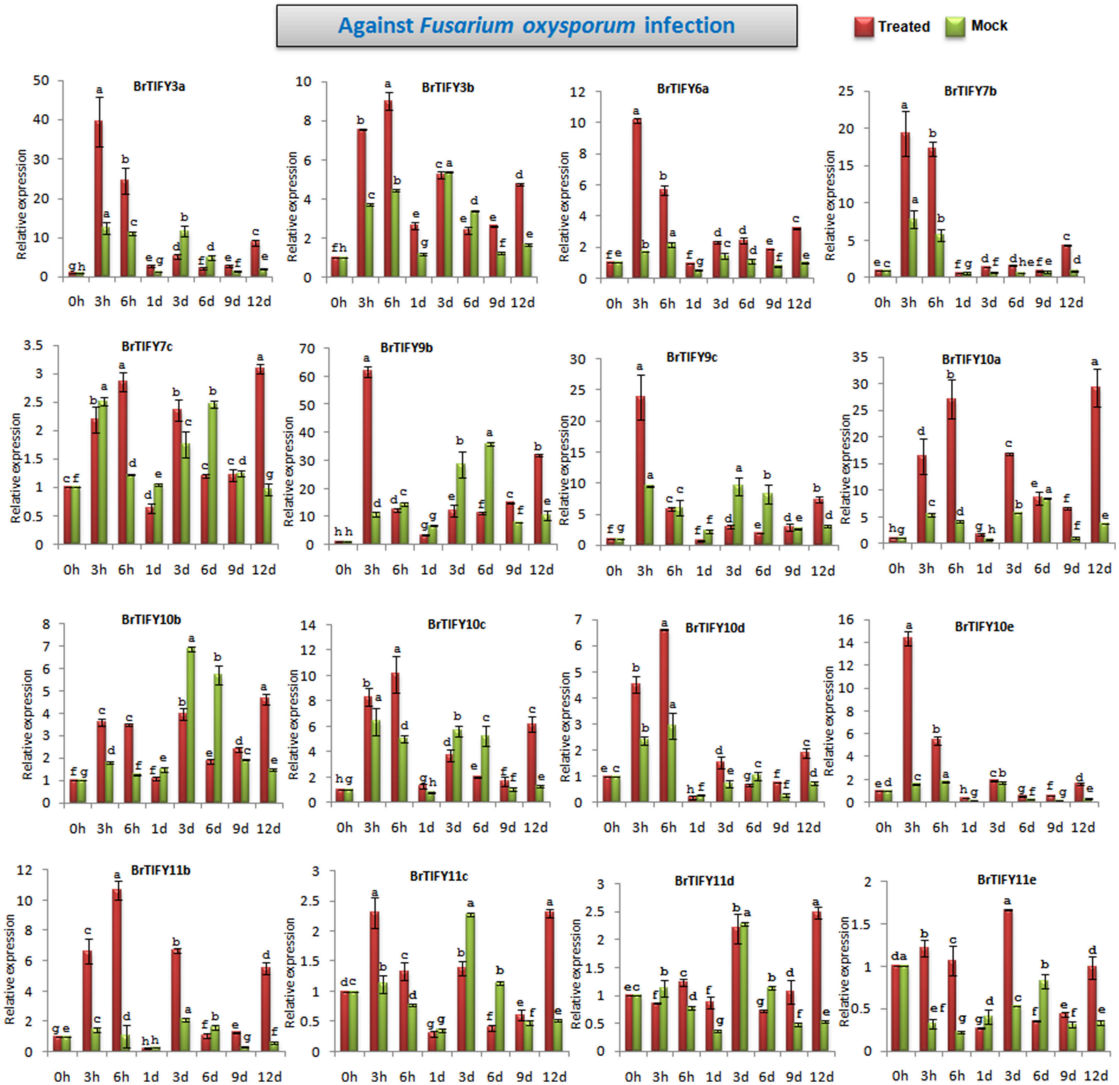
**Showing RT-qPCR expression analyses of 16 ***JAZ*** genes in ***B. rapa*** after infection with ***Fusarium oxysporum*** f.sp. ***conglutinans*** (0–12 d)**. The error bars represent the standard error of the means of three independent replicates. Values denoted by the same letter did not differ significantly at *P* < 0.05 according to Duncan's multiple range tests.

*TIFY* genes are involved in responses to different biotic elicitors in *Arabidopsis* and grape (Demianski et al., 2012; Zhang et al., 2012). Zhang et al. (2012) reported that *TIFY* genes in grape showed significant induction in response to fungal infection. In this study, we analyzed the expression of *JAZ* genes in response to *F. oxysporum* f.sp. *conglutinans*, which specifically attacks *Brassica* species and causes wilt and root rot diseases. After *Fusarium* infection of *B. rapa* Chiifu, we observed the responses of *JAZ* genes up to 12 days (Figure 10, Supplementary Figure 3). Among 21 *JAZ* genes, 16 showed several fold up-regulations during *Fusarium* infection compared to mock treatments. In general, most of the genes were strongly induced at early time points (3 and 6 h) and were down-regulated especially during the period from 1 to 9 d, followed by up-regulation at 12 d. *BrTIFY9b* showed the highest up-regulation, by >60-fold at 3 h compared to the control, which was 6-fold greater induction than that exhibited in the mock treatment (10-fold). The expression then decreased from 6 h to 9 d, followed by up-regulation again at 12 d by 3-fold compared to mock treatment. *BrTIFY9c* is a close homolog of *BrTIFY9b* and other members of the JAZ subfamily, *viz. BrTIFY3a, 3b, 6a, 7b, 10c, 10d*, and *10e*, also showed similar patterns of expression after *Fusarium* infection. Other JAZ members such as *BrTIFY10a, 10b, 11b, 11c, 11d*, and *11e* showed differential up-regulation throughout the infection period. Most of these genes had higher up-regulation by several fold during infection compared to mock treatment. Notably, this group of *JAZ* genes showed relatively higher expression at the later stage of infection (12 d).

In a nutshell, from the stress treatment experiments, we identified several *JAZ* genes showing up-regulation in response to different abiotic, biotic and hormone treatments that might be good candidates for developing stress resistance in *B. rapa*. In particular, *BrTIFY9a, 9b, 9c* and several genes from the *BrIFY10* and *BrIFY11* subgroups were strongly induced by most of the stresses.

#### Analysis of TIFY protein interaction networks in *B. rapa*

**Figure 11:**
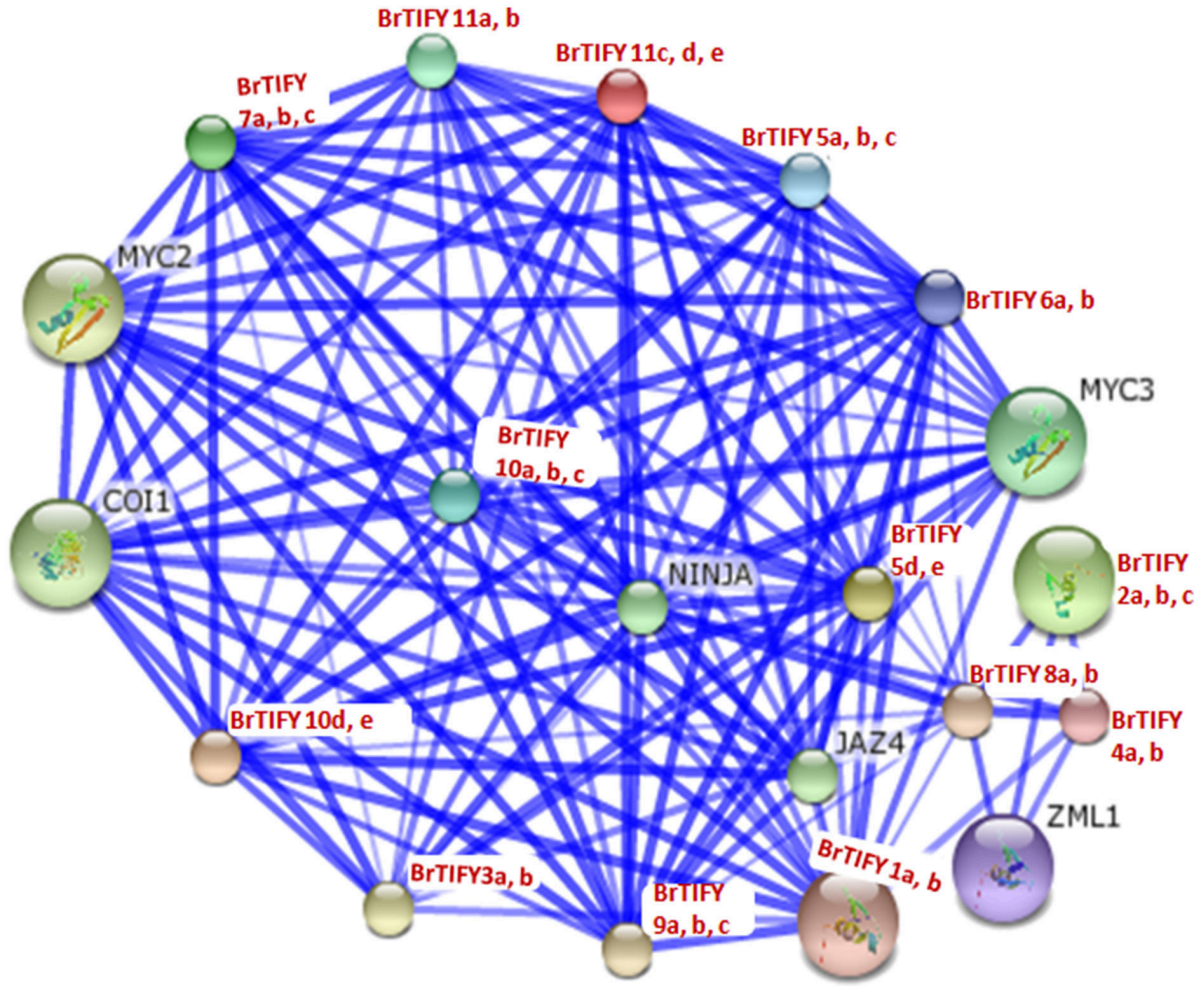
**Interaction network of 36 TIFY proteins identified in ***B. rapa*** with their predicted functional partners based on related proteins in Arabidopsis**. Stronger associations are represented by thicker lines.

We conducted a network interaction analysis for all 36 *B. rapa* TIFY proteins to predict their interactions and associations based on *Arabidopsis* proteins using STRING software (Figure 11). *Arabidopsis* CORONATINE INSENSITIVE 1 (COI1), NOVEL INTERACTOR OF JAZ (NINJA) and bHLH transcription factor family members MYC2 and MYC3, predicted to function in the processes of JA signaling in which they were supposed to interact with all of the *B. rapa* TIFY proteins. COI1, NINJA, and MYC2/MYC3 proteins have been reported to take part in JA signaling and different JA-dependent functions *viz*. root growth, plant fertility, biotic and abiotic stress resistance (Xie et al., 1998; Chini et al., 2007; Thines et al., 2007; Browse, 2009). We identified constitutive expression of *B. rapa* JAZ proteins in different tissues and in different growth stages during flower development. Most of these JAZ proteins were also found to be induced strongly in response to JA, ABA, environmental stresses including cold, salt, and drought and necrotrophic pathogen *Fusarium*. We speculate that these proteins might interact with COI1, NINJA, and MYC2/MYC3 in a JA-dependent manner for their functions, especially against different stresses and during flower development in *B. rapa*.

## Discussion

Members of the TIFY family have been demonstrated to be putative TFs with various stress responsiveness (Ye et al., 2009; Demianski et al., 2012; Zhang et al., 2012). Particularly, the JAZ subfamily genes are the best characterized as responsive to stresses to date (Li et al., 2015). However, there is no information regarding the expression and functions of this gene family in economically important crop species *B. rapa*, for which production is affected by various abiotic and biotic stresses. Hence, we conducted this genome-wide identification and characterization of *B. rapa TIFY* genes, including their tissue-specific expression in different organs and in response to different abiotic, biotic, and hormone treatments.

Specifically, we studied the expression patterns of JAZ subfamily genes in different tissues and found their widespread expression in flower buds (Figure 6). Subsequent analysis of these genes in different floral tissues and different flower growth stages also revealed their likely involvement in the development of *B. rapa* flowers. Members of the JAZ subfamily have been reported to play roles in flower induction (Kim et al., 2011), stamen development (Song et al., 2011), and defense responses against biotic (Shoji et al., 2008; Sun et al., 2011; Demianski et al., 2012; Oh et al., 2012) and abiotic (Ye et al., 2009; Seo et al., 2011; Ismail et al., 2012; Zhu et al., 2012) stresses. Specifically, these genes have been associated with JA responses, where jasmonates stimulate the Skp1-Cullin-F-box (SCF) E3 ubiquitin ligase complex SCF^COI1^ -mediated degradation of JAZ proteins to derepress transcription factors like MYC2 and MYC3 (Yan et al., 2007; Melotto et al., 2008; Bai et al., 2011). In this mechanism, up-regulation of certain *JAZ* genes is considered to be a method of fine-tuning JA responses (Chung et al., 2009).

Rice *JAZ* genes are induced by abiotic stresses like low temperature, drought and salinity, and *OsTIFY11a* was found to improve stress tolerance (Ye et al., 2009). In grape, 11 *TIFY* genes were found responsive to osmotic and cold stresses (Zhang et al., 2012). In our study, we found JAZ subfamily genes that showed differential up-regulation against cold, salt and drought treatments in *B. rapa*. From low temperature-treated whole-genome microarray data and subsequent validation via qPCR, we identified some good candidate *B. rapa JAZ* genes (*BrTIFY3a, 6a, 6b, 7b, 9b, 9c, 10c, 10f, 11a*, and *11c*) to be involved in response to low temperatures. This group of genes was also implicated in salinity and drought stress response, during which they showed stronger induction than under cold stress. More specifically, these genes had the highest up-regulation in response to salt stress among three abiotic stresses (in case of *BrTIFY9a, 9b*, and *9c*; Figures 7, 8).

In response to hormone treatments, *B. rapa JAZ* genes were highly regulated by JA and ABA, but not by SA. Several studies have been demonstrated that JA treatment and environmental cues rapidly trigger *JAZ* gene expression, which might be responsible for moderating the response to JA (Chini et al., 2007; Thines et al., 2007; Katsir et al., 2008a). Functional evidence shows that, in response to different phytohormones, *JAZ* genes seem to have wider regulatory roles in various aspects of plant development than those performed by other subfamily genes of TIFY (Zhang et al., 2012). In this study, we found that all *B. rapa JAZ* genes were strongly induced and showed high up-regulation (by >15-fold to >800-fold compared to the reference), especially at the final time point (48 h) of JA treatment (Figure 9A). However, against SA, 15 genes showed up-regulation at lower levels (maximum >5 fold) and the rest of the genes were down-regulated and became inactive (Figure 9B). ABA plays key roles in helping the plant to adjust to adverse environmental conditions like drought, cold and salinity (Davies and Jones, 1991). For instance, 20 rice *TIFY* genes exhibited strong induction in response to ABA and most of these genes were also induced by abiotic stresses (Ye et al., 2009). In our analysis, most of the abiotic stress-responsive *JAZ* genes were also induced by exogenous ABA treatment. Only three genes (*BrTIFY10a, 11a*, and *11b*) were seemingly not regulated by ABA (Figure 9C). It is possible that both ABA-dependent and ABA-independent signaling pathways are responsible for regulating the expression of *B. rapa JAZ* genes.

*JAZ* genes play roles against different biotic agents like insect herbivory and pathogen infection (Shoji et al., 2008; Sun et al., 2011; Demianski et al., 2012; Oh et al., 2012) and many of these resistance mechanisms are mediated by a variety of signaling molecules that include JA, SA, and ET (ethylene) (Bari and Jones, 2009; Pieterse et al., 2009). Jasmonates might play roles in plant resistance against necrotrophic pathogens like *Alternaria brassicicola* and *Botrytis cinerea* (Glazebrook, 2005). In this study, the majority of the *JAZ* genes (16 out of 21 genes) showed responsiveness to the necrotrophic pathogen *F. oxysporum* in the form of differential up-regulation of their transcript levels (Figure 10). Notably, several genes exhibited up-regulation during the early period (3 and 6 h) of infection (*BrTIFY3a, 3b, 6a, 7b, 9b, 9c, 10c, 10d*, and *10e*). Another set of *JAZ* genes (*BrTIFY10a, 10b, 11b, 11c, 11d*, and *11e*) showed differential up-regulation throughout the infection period (3 h–12 d), with the highest expression at 12 d. Thus, both groups of genes are considered to be responsive at differential levels to *Fusarium* infection in *B. rapa*. This phenomenon may be explained by the fact that expression of *B. rapa JAZ* genes could be induced by a necrotrophic pathogen like *Fusarium* through triggering activation of JA-dependent defense responses. In contrary, there is some previous evidence that *JAZ* genes are induced by biotrophic pathogens. For instance, *Arabidopsis JAZ* genes showed up-regulation in response to the biotrophic pathogen *Pseudomonas syringae*. Generally, though, the SA-dependent pathway is considered to be the regulating factor in activation of defense responses to biotrophic infection (Glazebrook, 2005). However, up-regulation of *Arabidopsis JAZ* following infection with *P. syringae* might be related to the fact that the virulence factor coronatine is an inducer of JA/ET signaling (Glazebrook, 2005; Demianski et al., 2012).

Furthermore, we may look for some intersecting points among the signaling pathways of JA, ABA, and SA. Expression of genes in response to stresses might converge or interact in some points of the signaling pathways. For example, ABA and JA have been reported to act synergistically during drought stress signaling. The overlapping signaling cascade of ABA and JA certainly needs a common player to interact. MYC2 has been identified to play roles for multiple hormone signaling pathways (Riemann et al., 2015). It has also been reported that TF MYC2 activates downstream genes like *RD22/RD26* whose expression is induced by pathogens, drought, salt, ABA, JA treatment (Fujita et al., 2004, 2006; Harb et al., 2010). In our study, *JAZ* genes were induced by multiple treatments like JA, ABA, SA as well as by abiotic stresses including low temperature, drought, salinity, and pathogen infection. We may postulate that *JAZ* genes in *B. rapa* responded against different stresses following different hormone signaling pathways and TFs like MYC2/3/4 might be a common interacting point to regulate different downstream genes.

In conclusion, among the four TIFY subfamilies, JAZ proteins have been characterized extensively as key factors in JA signaling, whereas less is known about the other subfamilies. However, remarkable progress in the identification and characterization of TIFY family genes in different higher plants has been made using functional evidence regarding these genes in the model plant *Arabidopsis*. Our current comprehensive and systemic analysis of *TIFY* genes in the *B. rapa* genome identified seven *TIFY*, six *ZML*, two *PPD* and 21 *JAZ* genes. The JAZ family genes in *B. rapa* Chiifu were found to be induced by low temperature more highly than members of the other subfamilies. The *JAZ* genes had constitutive expression patterns, mainly in the reproductive parts and during flower development in *B. rapa*. We further identified potential involvement of most of the *JAZ* genes in tolerance to cold, salt and drought. *B. rapa JAZ* genes were highly induced by JA/ABA and moderately by SA. *JAZ* genes were also induced strongly in response to the necrotrophic fungal pathogen *Fusarium*. Taken as a whole, our study identified several *B. rapa JAZ* genes (*BrTIFY3a, 3b, 6a, 9a, 9b*, and *9c*) that showed corresponsive expression against all the stresses and phytohormone treatments. This study thus supplements our understanding of this gene family in plants and provides the basis for future functional studies on *TIFY* genes in *B. rapa*.

## Author contributions

IN, JP planned the work and provided suggestions to organize data to first author and also provided editorial comments. GS designed and conducted all experiments under the supervision of IN, JP. MK assisted GS with experimentation and data collection. All authors read and agreed the manuscript contents.

### Conflict of interest statement

The authors declare that the research was conducted in the absence of any commercial or financial relationships that could be construed as a potential conflict of interest.
