# Supplementary material for: A Genome-Wide Analysis Reveals Stress and Hormone Responsive Patterns of TIFY Family Genes in Brassica rapa
Source: Front Plant Sci. 2016 Jun 28;7:936. doi: 10.3389/fpls.2016.00936 (PMC4923152; doi:10.3389/fpls.2016.00936)
Supplement: Supplementary file 1 [file Table1.DOC]

**Supplementary Table 1 │** List of *B. rapa TIFY* primers used for RT-PCR and qPCR analysis

| **Gene name** | **Left primer** | **Right primer** | **Product length** |
| --- | --- | --- | --- |
| BrTIFY3a | CTCTCTCAAGCACATCCAAT | GATCTTCTGCTAAGCAGTC | 248 |
| BrTIFY3b | AGTTTGTGTCTTCGATGGAA | GCAAAACGGAATTGGGTTAA | 214 |
| BrTIFY6a | CGAGTAGAGGAATGAAGTGG | TCCTAACTCCCTGAACAGAA | 213 |
| BrTIFY6b | TAGCACAAATGTACTCGGAG | TGCATTCAGATATTGGCGTA | 221 |
| BrTIFY7a | GCGACTAATGCAATGAGCAT | GCCTTTTAGGTATGAGAAGC | 218 |
| BrTIFY7b | AGAAGGCAGCTATACAATGG | AGAAGATAGTGAGCTGAGGT | 276 |
| BrTIFY7c | CGGAACTGTTACCGTCTTTA | CATATTGCTAGGACCTGCAT | 289 |
| BrTIFY9a | CAGATCAAAGATGTCTAGAG | TAATCTGCCTTTGGAGTAG | 246 |
| BrTIFY9b | CCAAACATGTCTAAGGTTAC | CGATAGAGATCTGGTCGAGG | 216 |
| BrTIFY9c | CTACAATGGAACCGTCTC | AGCAATTATCTCTCCTTGCG | 237 |
| BrTIFY10a | GAAGTTACCTGAGAAGCCAA | GCTTCACAGGTTTAGTCTCT | 248 |
| BrTIFY10b | AAATCCAGTTCCTAGCCTTG | CAAGATGTGTCAGGCCTGGAAG | 204 |
| BrTIFY10c | GAGATTGCTTCCACTACTCC | CTGTGTTAGGCTTGGAAGAT | 217 |
| BrTIFY10d | GTTAAAATGATGTCAAGTTC | CCTAGGAAACAGATTCTTG | 255 |
| BrTIFY10e | GTATAACGATGTCGAGTCC | GCTTCACAAGGGAATAAACTC | 217 |
| BrTIFY10f | TCTCAAGACCCAAAGCAAAC | CGATATCACGTCACATATC | 220 |
| BrTIFY11a | ATCATCAAACAGGACAGCAA | TCTCTTTGGTTTTGAGGGAG | 221 |
| BrTIFY11b | GTATCATCACAGTATGTCGAC | ATGTTTGCCTTCTCTATTGC | 198 |
| BrTIFY11c | GCTAAACGTAAAGACAGGGCTG | TTTGATTATAGCCTAAGTTCG | 236 |
| BrTIFY11d | GTGGGTCAGCATCATTATCC | ACTTTACTATAGCCTAAGC | 201 |
| BrTIFY11e | AGCCAAAGAGATCATGGAAG | GATTTTCTTGTTGCTGCTGT | 215 |
