# Supplementary material for: A Genome-Wide Analysis Reveals Stress and Hormone Responsive Patterns of TIFY Family Genes in Brassica rapa
Source: Front Plant Sci. 2016 Jun 28;7:936. doi: 10.3389/fpls.2016.00936 (PMC4923152; doi:10.3389/fpls.2016.00936)
Supplement: Supplementary file 3 [file Table3.DOC]

**Supplementary Table 3 │** Homology analysis of 36 *B. rapa* TIFY proteins

| **Sl.** | **Protein name** | **Top matched clones** | **Name of homolog protein** | **Identity** | ***E*-value** | **Top homologous species** | **References** |
| --- | --- | --- | --- | --- | --- | --- | --- |
| 1. | BrTIFY1a | [NP_194178](http://www.ncbi.nlm.nih.gov/protein/15233844?report=genbank&log$=prottop&blast_rank=4&RID=K5FV1J98014) | putative GATA transcription factor | 82% | 2.00E-155 | *A. thaliana* | Mayer *et al.* (1999) |
| 2. | BrTIFY1b | [BAA97678](http://www.ncbi.nlm.nih.gov/protein/8918531?report=genbank&log$=prottop&blast_rank=3&RID=K5KG8KCJ01R) | [ZIM](http://blast.ncbi.nlm.nih.gov/Blast.cgi#alnHdr_8918531) | 69% | 6.00E-125 | *A. thaliana* | Nishii *et al.* (2000) |
| 3. | BrTIFY1c | [BAA97678](http://www.ncbi.nlm.nih.gov/protein/8918531?report=genbank&log$=prottop&blast_rank=6&RID=K6ZD9B5501R) | [ZIM](http://blast.ncbi.nlm.nih.gov/Blast.cgi#alnHdr_8918531) | 71% | 7.00E-40 | *A. thaliana* | Nishii *et al.* (2000) |
| 4. | BrTIFY2a | [NP_564593](http://www.ncbi.nlm.nih.gov/protein/18403600?report=genbank&log$=prottop&blast_rank=3&RID=K5G4Y83W014) | [GATA transcription factor 28](http://blast.ncbi.nlm.nih.gov/Blast.cgi#alnHdr_18403600) | 84% | 3.00E-157 | *A. thaliana* | Theologis *et al.* (2000) |
| 5. | BrTIFY2b | [NP_850618](http://www.ncbi.nlm.nih.gov/protein/30686115?report=genbank&log$=prottop&blast_rank=3&RID=K7115CR801R) | [GATA transcription factor 24]](http://blast.ncbi.nlm.nih.gov/Blast.cgi#alnHdr_30686115) | 72% | 3.00E-156 | *A. thaliana* | Salanoubat *et al.* (2000) |
| 6. | BrTIFY2c | [NP_850618](http://www.ncbi.nlm.nih.gov/protein/30686115?report=genbank&log$=prottop&blast_rank=2&RID=K7179RZ401R) | [GATA transcription factor 24](http://blast.ncbi.nlm.nih.gov/Blast.cgi#alnHdr_30686115) | 79% | 1.00E-149 | *A. thaliana* | Salanoubat *et al.* (2000) |
| 7. | BrTIFY3a | [NP_197590](http://www.ncbi.nlm.nih.gov/protein/15242096?report=genbank&log$=prottop&blast_rank=3&RID=K4SK22YM015) | [protein TIFY 3B](http://blast.ncbi.nlm.nih.gov/Blast.cgi#alnHdr_15242096) | 65% | 2.00E-79 | *A. thaliana* | Unpublished |
| 8. | BrTIFY3b | [NP_197590](http://www.ncbi.nlm.nih.gov/protein/15242096?report=genbank&log$=prottop&blast_rank=3&RID=K6VR41B601R) | [protein TIFY 3B](http://blast.ncbi.nlm.nih.gov/Blast.cgi#alnHdr_15242096) | 61% | 7.00E-77 | *A. thaliana* | Unpublished |
| 9. | BrTIFY4a | [NP_193208](http://www.ncbi.nlm.nih.gov/protein/30682967?report=genbank&log$=prottop&blast_rank=2&RID=K70H4JB501R) | [protein PEAPOD2](http://blast.ncbi.nlm.nih.gov/Blast.cgi#alnHdr_30682967) | 79% | 5.00E-173 | *A. thaliana* | Mayer *et al.* (1999) |
| 10. | BrTIFY4b | [NP_193208](http://www.ncbi.nlm.nih.gov/protein/30682967?report=genbank&log$=prottop&blast_rank=1&RID=K70P1RD501R) | [protein PEAPOD2](http://blast.ncbi.nlm.nih.gov/Blast.cgi#alnHdr_30682967) | 76% | 2.00E-156 | *A. thaliana* | Mayer *et al.* (1999) |
| 11. | BrTIFY5a | [NP_181007](http://www.ncbi.nlm.nih.gov/protein/15226783?report=genbank&log$=prottop&blast_rank=1&RID=K4TZKH6V014) | [jasmonate-zim-domain protein 7](http://blast.ncbi.nlm.nih.gov/Blast.cgi#alnHdr_15226783) | 64% | 4.00E-40 | *A. thaliana* | Lin *et al.* (1999) |
| 12. | BrTIFY5b | [NP_181007](http://www.ncbi.nlm.nih.gov/protein/15226783?report=genbank&log$=prottop&blast_rank=1&RID=K6WJBKTF01R) | [jasmonate-zim-domain protein 7](http://blast.ncbi.nlm.nih.gov/Blast.cgi#alnHdr_15226783) | 66% | 9.00E-44 | *A. thaliana* | Lin *et al.* (1999) |
| 13. | BrTIFY5c | [NP_181007](http://www.ncbi.nlm.nih.gov/protein/15226783?report=genbank&log$=prottop&blast_rank=1&RID=K6WTM38301R) | [jasmonate-zim-domain protein 7](http://blast.ncbi.nlm.nih.gov/Blast.cgi#alnHdr_15226783) | 60% | 7.00E-33 | *A. thaliana* | Lin *et al.* (1999) |
| 14. | BrTIFY5d | [NP_564349](http://www.ncbi.nlm.nih.gov/protein/18397376?report=genbank&log$=prottop&blast_rank=2&RID=K54WEEU9015) | [protein TIFY 5A](http://blast.ncbi.nlm.nih.gov/Blast.cgi#alnHdr_18397376) | 77% | 1.00E-60 | *A. thaliana* | Theologis *et al.* (2000) |
| 15. | BrTIFY5e | [NP_564349](http://www.ncbi.nlm.nih.gov/protein/18397376?report=genbank&log$=prottop&blast_rank=2&RID=K6ZRJMT501R) | [protein TIFY 5A](http://blast.ncbi.nlm.nih.gov/Blast.cgi#alnHdr_18397376) | 69% | 3.00E-51 | *A. thaliana* | Theologis *et al.* (2000) |
| 16. | BrTIFY6a | [NP_566590](http://www.ncbi.nlm.nih.gov/protein/18401669?report=genbank&log$=prottop&blast_rank=2&RID=K6W4G6RU01R) | [jasmonate-zim-domain protein 3](http://blast.ncbi.nlm.nih.gov/Blast.cgi#alnHdr_18401669) | 83% | 0.0 | *A. thaliana* | Salanoubat *et al.* (2000) |
| 17. | BrTIFY6b | [NP_566590](http://www.ncbi.nlm.nih.gov/protein/18401669?report=genbank&log$=prottop&blast_rank=2&RID=K6WNBDR001R) | [jasmonate-zim-domain protein 3](http://blast.ncbi.nlm.nih.gov/Blast.cgi#alnHdr_18401669) | 81% | 0.0 | *A. thaliana* | Salanoubat *et al.* (2000) |
| 18. | BrTIFY7a | [NP_177227](http://www.ncbi.nlm.nih.gov/protein/240254344?report=genbank&log$=prottop&blast_rank=2&RID=K4TTAJB6015) | [putative jasmonate signaling protein JAZ9](http://blast.ncbi.nlm.nih.gov/Blast.cgi#alnHdr_240254344) | 57% | 1.00E-85 | *A. thaliana* | Theologis *et al.* (2000) |
| 19. | BrTIFY7b | [NP_177227](http://www.ncbi.nlm.nih.gov/protein/240254344?report=genbank&log$=prottop&blast_rank=3&RID=K4UZRFK8015) | [putative jasmonate signaling protein JAZ9](http://blast.ncbi.nlm.nih.gov/Blast.cgi#alnHdr_240254344) | 72% | 2.00E-107 | *A. thaliana* | Theologis *et al.* (2000) |
| 20. | BrTIFY7c | [NP_177227](http://www.ncbi.nlm.nih.gov/protein/240254344?report=genbank&log$=prottop&blast_rank=2&RID=K5GNK0W701R) | [putative jasmonate signaling protein JAZ9](http://blast.ncbi.nlm.nih.gov/Blast.cgi#alnHdr_240254344) | 67% | 4.00E-99 | *A. thaliana* | Theologis *et al.* (2000) |
| 21. | BrTIFY8a | [NP_567898](http://www.ncbi.nlm.nih.gov/protein/18418038?report=genbank&log$=prottop&blast_rank=2&RID=K5506WWZ014) | [protein TIFY 8](http://blast.ncbi.nlm.nih.gov/Blast.cgi#alnHdr_18418038) | 76% | 2.00E-163 | *A. thaliana* | Mayer *et al.* (1999) |
| 22. | BrTIFY8b | [NP_567898](http://www.ncbi.nlm.nih.gov/protein/18418038?report=genbank&log$=prottop&blast_rank=3&RID=K70SZDCJ01R) | [protein TIFY 8](http://blast.ncbi.nlm.nih.gov/Blast.cgi#alnHdr_18418038) | 83% | 0.0 | *A. thaliana* | Mayer *et al.* (1999_ |
| 23. | BrTIFY9a | [NP_568287](http://www.ncbi.nlm.nih.gov/protein/18416997?report=genbank&log$=prottop&blast_rank=3&RID=K4UWR7MT014) | [protein TIFY 9](http://blast.ncbi.nlm.nih.gov/Blast.cgi#alnHdr_18416997) | 74% | 2.00E-92 | *A. thaliana* | Unpublished |
| 24. | BrTIFY9b | [NP_568287](http://www.ncbi.nlm.nih.gov/protein/18416997?report=genbank&log$=prottop&blast_rank=4&RID=K54TAZBZ014) | [protein TIFY 9](http://blast.ncbi.nlm.nih.gov/Blast.cgi#alnHdr_18416997) | 79% | 3.00E-102 | *A. thaliana* | Unpublished |
| 25. | BrTIFY9c | [NP_974775](http://www.ncbi.nlm.nih.gov/protein/42573357?report=genbank&log$=prottop&blast_rank=4&RID=K6XEC7AY01R) | [protein TIFY 9](http://blast.ncbi.nlm.nih.gov/Blast.cgi#alnHdr_42573357) | 77% | 3.00E-93 | *A. thaliana* | Unpublished |
| 26. | BrTIFY10a | [NP_565096](http://www.ncbi.nlm.nih.gov/protein/18410737?report=genbank&log$=prottop&blast_rank=3&RID=K4TNGRR3015) | [protein TIFY 10B](http://blast.ncbi.nlm.nih.gov/Blast.cgi#alnHdr_18410737) | 65% | 4.00E-86 | *A. thaliana* | Theologis *et al.* (2000) |
| 27. | BrTIFY10b | [NP_565096](http://www.ncbi.nlm.nih.gov/protein/18410737?report=genbank&log$=prottop&blast_rank=4&RID=K54N24W1014) | [protein TIFY 10B](http://blast.ncbi.nlm.nih.gov/Blast.cgi#alnHdr_18410737) | 68% | 1.00E-94 | *A. thaliana* | Theologis *et al.* (2000) |
| 28. | BrTIFY10c | [NP_565096](http://www.ncbi.nlm.nih.gov/protein/18410737?report=genbank&log$=prottop&blast_rank=3&RID=K5G8VSFC014) | [protein TIFY 10B](http://blast.ncbi.nlm.nih.gov/Blast.cgi#alnHdr_18410737) | 71% | 1.00E-102 | *A. thaliana* | Theologis *et al.* (2000) |
| 29. | BrTIFY10d | [NP_564075](http://www.ncbi.nlm.nih.gov/protein/18394706?report=genbank&log$=prottop&blast_rank=2&RID=K5H565GS01R) | [jasmonate-zim-domain protein 1](http://blast.ncbi.nlm.nih.gov/Blast.cgi#alnHdr_18394706) | 72% | 1.00E-113 | *A. thaliana* | Theologis *et al.* (2000) |
| 30. | BrTIFY10e | [NP_564075](http://www.ncbi.nlm.nih.gov/protein/18394706?report=genbank&log$=prottop&blast_rank=2&RID=K6Z9UTB601R) | [jasmonate-zim-domain protein 1](http://blast.ncbi.nlm.nih.gov/Blast.cgi#alnHdr_18394706) | 74% | 6.00E-127 | *A. thaliana* | Theologis *et al.* (2000) |
| 31. | BrTIFY10f | [NP_564075](http://www.ncbi.nlm.nih.gov/protein/18394706?report=genbank&log$=prottop&blast_rank=3&RID=K6XHMR9001R) | [jasmonate-zim-domain protein 1](http://blast.ncbi.nlm.nih.gov/Blast.cgi#alnHdr_18394706) | 73% | 5.00E-112 | *A. thaliana* | Theologis *et al.* (2000) |
| 32. | BrTIFY11a | [NP_565043](http://www.ncbi.nlm.nih.gov/protein/18410088?report=genbank&log$=prottop&blast_rank=2&RID=K54FA3JN015) | [jasmonate-zim-domain protein 6](http://blast.ncbi.nlm.nih.gov/Blast.cgi#alnHdr_18410088) | 72% | 6.00E-132 | *A. thaliana* | Theologis *et al.* (2000) |
| 33. | BrTIFY11b | [NP_565043](http://www.ncbi.nlm.nih.gov/protein/18410088?report=genbank&log$=prottop&blast_rank=2&RID=K5GC3XYH01R) | [jasmonate-zim-domain protein 6](http://blast.ncbi.nlm.nih.gov/Blast.cgi#alnHdr_18410088) | 73% | 3.00E-135 | *A. thaliana* | Theologis *et al.* (2000) |
| 34. | BrTIFY11c | [NP_564019](http://www.ncbi.nlm.nih.gov/protein/18394473?report=genbank&log$=prottop&blast_rank=3&RID=K5JDAMV301R) | [protein TIFY 11A](http://blast.ncbi.nlm.nih.gov/Blast.cgi#alnHdr_18394473) | 67% | 1.00E-107 | *A. thaliana* | Theologis *et al.* (2000) |
| 35. | BrTIFY11d | [NP_564019](http://www.ncbi.nlm.nih.gov/protein/18394473?report=genbank&log$=prottop&blast_rank=2&RID=K6XMSH9401R) | [protein TIFY 11A](http://blast.ncbi.nlm.nih.gov/Blast.cgi#alnHdr_18394473) | 63% | 3.00E-100 | *A. thaliana* | Theologis *et al.* (2000) |
| 36. | BrTIFY11e | [NP_564019](http://www.ncbi.nlm.nih.gov/protein/18394473?report=genbank&log$=prottop&blast_rank=2&RID=K6XVYMN801R) | [protein TIFY 11A](http://blast.ncbi.nlm.nih.gov/Blast.cgi#alnHdr_18394473) | 68% | 2.00E-120 | *A. thaliana* | Theologis *et al.* (2000) |
