# Supplementary material for: A Genome-Wide Analysis Reveals Stress and Hormone Responsive Patterns of TIFY Family Genes in Brassica rapa
Source: Front Plant Sci. 2016 Jun 28;7:936. doi: 10.3389/fpls.2016.00936 (PMC4923152; doi:10.3389/fpls.2016.00936)
Supplement: Supplementary file 4 [file Presentation1.PPT]

## Slide 1
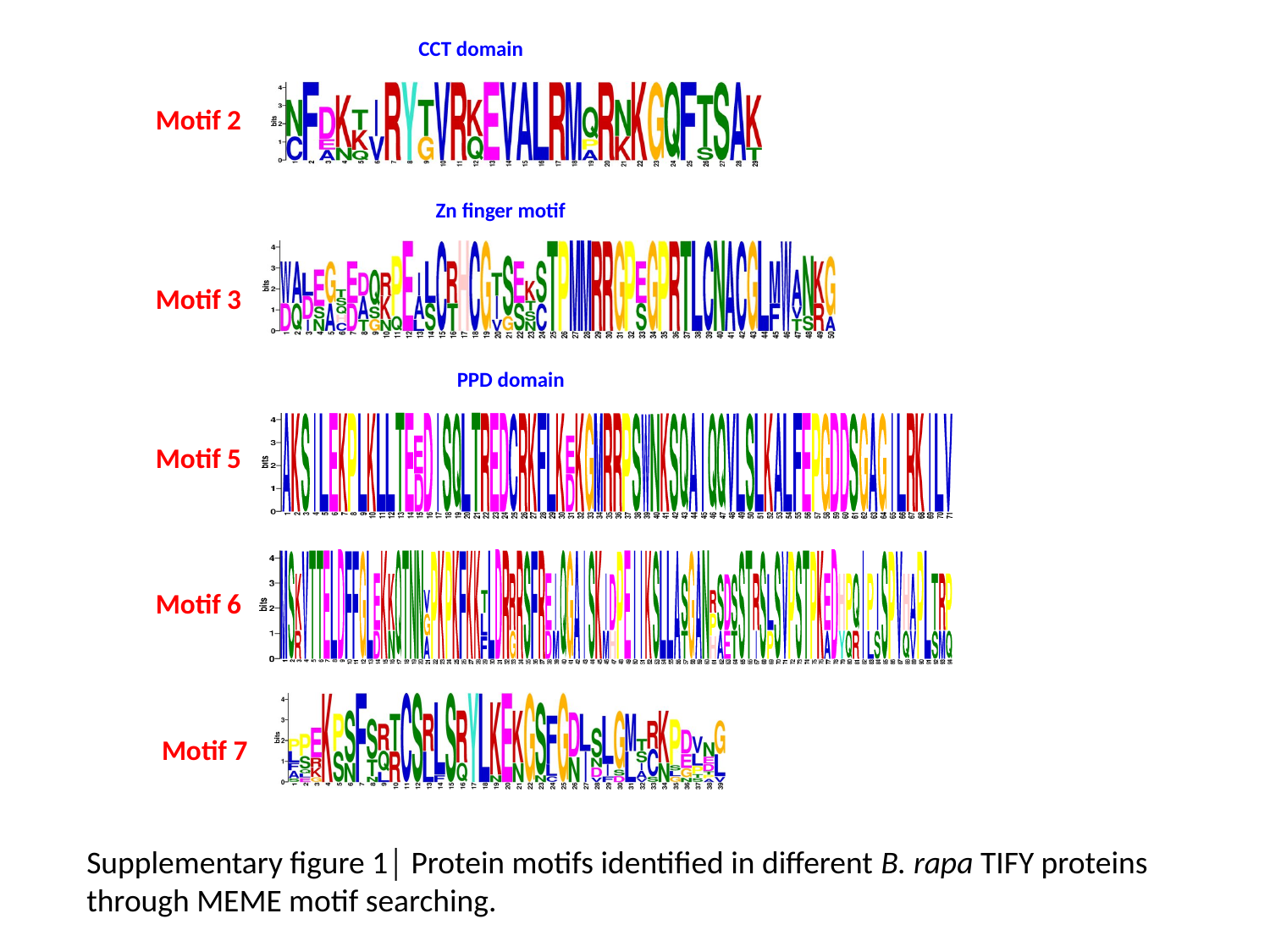

CCT domain
Motif 2
Zn finger motif
Motif 3
PPD domain
Motif 5
Motif 6
Motif 7
Supplementary figure 1│ Protein motifs identified in different B. rapa TIFY proteins through MEME motif searching.
