# Supplementary material for: A Genome-Wide Analysis Reveals Stress and Hormone Responsive Patterns of TIFY Family Genes in Brassica rapa
Source: Front Plant Sci. 2016 Jun 28;7:936. doi: 10.3389/fpls.2016.00936 (PMC4923152; doi:10.3389/fpls.2016.00936)
Supplement: Supplementary file 5 [file Presentation2.PPT]

## Slide 1
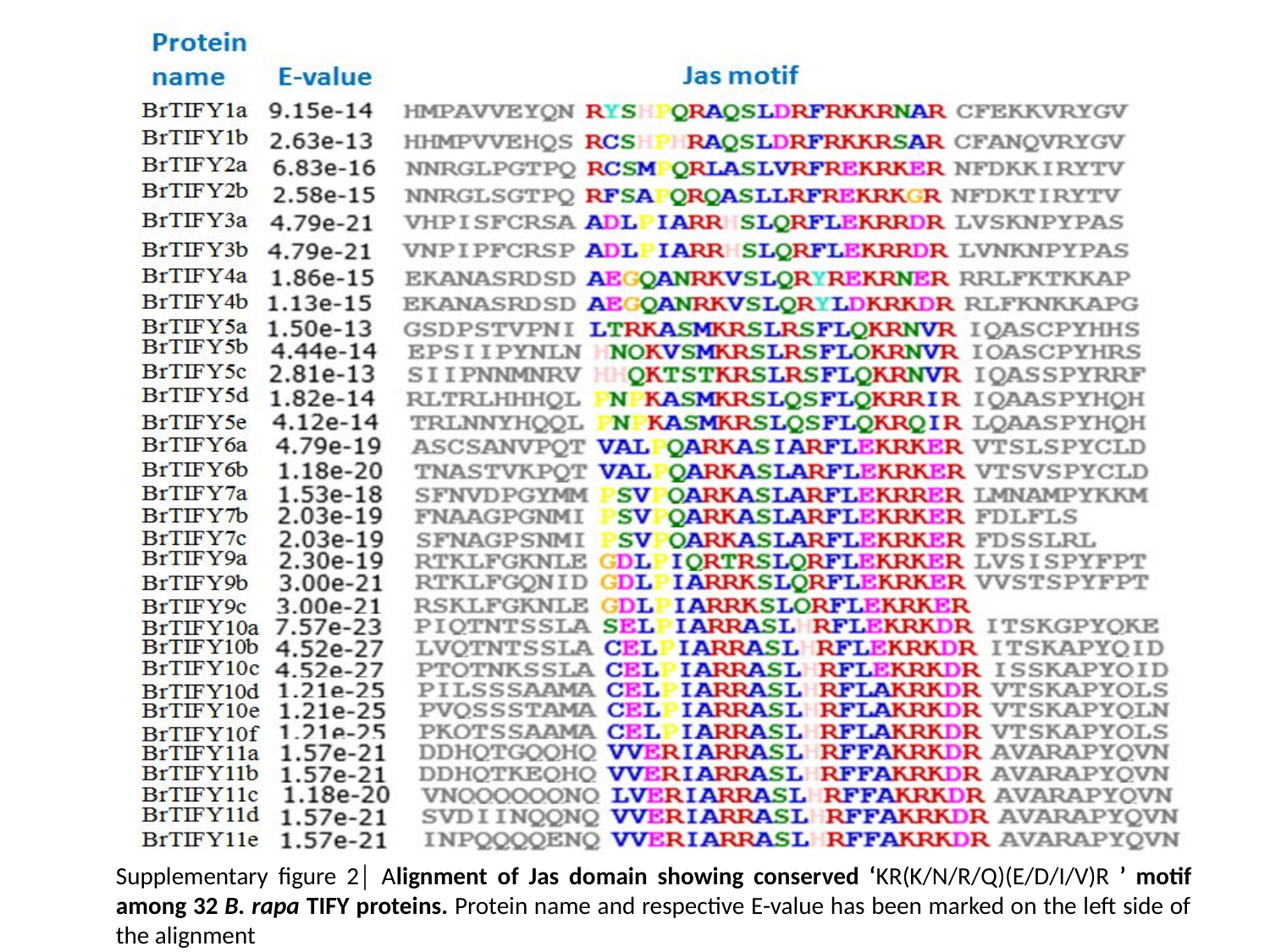

Supplementary figure 2│ Alignment of Jas domain showing conserved ‘KR(K/N/R/Q)(E/D/I/V)R ’ motif among 32 B. rapa TIFY proteins. Protein name and respective E-value has been marked on the left side of the alignment
