# Supplementary material for: A Genome-Wide Analysis Reveals Stress and Hormone Responsive Patterns of TIFY Family Genes in Brassica rapa
Source: Front Plant Sci. 2016 Jun 28;7:936. doi: 10.3389/fpls.2016.00936 (PMC4923152; doi:10.3389/fpls.2016.00936)
Supplement: Supplementary file 6 [file Presentation3.PPT]

## Slide 1
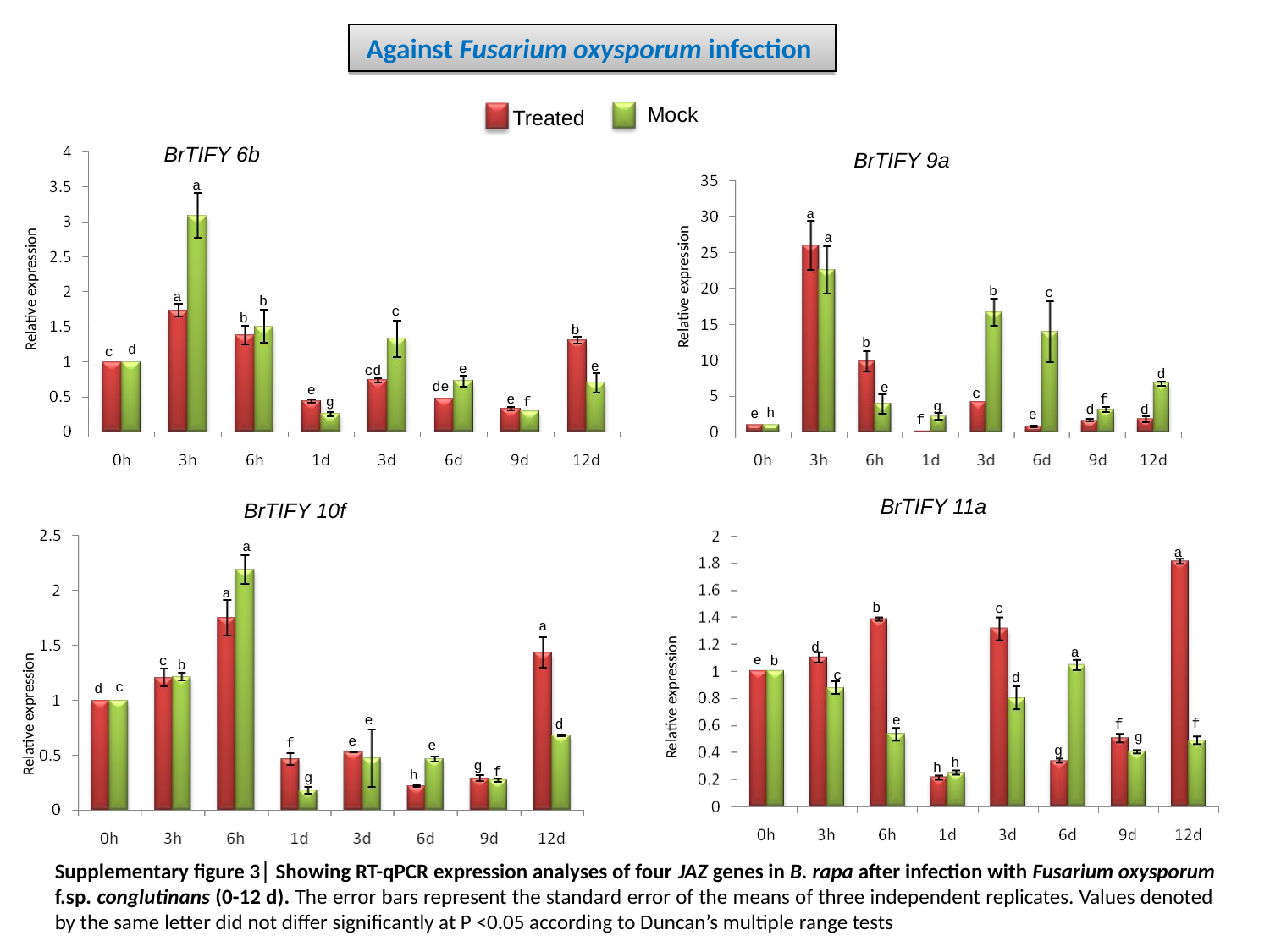

Against Fusarium oxysporum infection
Mock
Treated
a
b
c
b
b
d
c
e
e
cd
de
e
e
g
f
BrTIFY 6b
BrTIFY 9a
a
a
a
Relative expression
Relative expression
b
c
b
d
e
c
f
g
d
d
h
e
e
f
BrTIFY 11a
BrTIFY 10f
a
a
a
b
c
a
d
a
e
c
b
b
c
d
c
d
Relative expression
Relative expression
e
e
f
d
f
g
e
f
e
g
h
g
h
f
h
g
Supplementary figure 3│ Showing RT-qPCR expression analyses of four JAZ genes in B. rapa after infection with Fusarium oxysporum f.sp. conglutinans (0-12 d). The error bars represent the standard error of the means of three independent replicates. Values denoted by the same letter did not differ significantly at P <0.05 according to Duncan’s multiple range tests
